# The Use of Non-verbal Displays in Framing COVID-19 Disinformation in Europe: An Exploratory Account

**DOI:** 10.3389/fpsyg.2022.846250

**Published:** 2022-03-14

**Authors:** Delia Dumitrescu, Mina Trpkovic

**Affiliations:** Institute of Political Science, Heidelberg University, Heidelberg, Germany

**Keywords:** facial emotional expressions, body poses, COVID-19, disinformation, framing

## Abstract

While online disinformation practices have grown exponentially over the past decade, the COVID-19 pandemic provides arguably the best opportunity to date to study such communications at a cross-national level. Using the data provided by the International Fact-Checking Network (IFCN), we examine the strategic uses of non-verbal and verbal arguments to push disinformation through social media and websites during the first wave of lockdowns in 2020 across 16 European countries. Our paper extends the work by Brennen et al. (2021) on the use of visuals in COVID-19 misinformation claims by investigating the use of facial emotional expressions and body pose depictions in conjunction with framing elements such as problems identified and attribution of responsibility in the construction of disinformation messages. Our European-wide comparative analysis of 174 messages indexed by the IFCN during the months of April and May 2020 helps provide a rounder understanding of the use of non-verbal devices in advancing COVID-19 disinformation across the continent, and can provide the basis for a framework for further study of the strategic use of non-verbal devices in COVID-19 disinformation world-wide.

## Introduction

Disinformation and the ease of its dissemination through social media, are among the most significant global challenges today. The circulation of fake or misleading messages predates the COVID-19 pandemic (e.g., see Freelon and Wells, 2020; Scardigno and Minnini, 2020; Kapantai et al., 2021), but this multiyear, worldwide crisis provides a unique opportunity for understanding both the diversity and the similarities in the crafting of disinformation messages, and how they may change through time.

This study focuses on the contribution of non-verbal displays to the design of disinformation messages, by examining the patterns of association between, on the one hand, depictions of positive and negative facial emotional displays and contractive and expansive body poses, and framing elements in the disinformation posts—topics, the problems identified and the attribution of blame—on the other hand. We also examine the association between these non-verbal displays and the type of individuals pictured in the posts, whether human exemplars, standing for ordinary people, or the rich and powerful—such as politicians, businesspeople, and experts.

Our aim is exploratory. We make use of the database of COVID-19 disinformation messages put together by the International Fact-Checking Network (IFCN) at the Poynter Institute to examine how dual verbal-visual messages were constructed during the first fully-fledged lockdown in April-May 2020 in 16 European countries. While building on previous research (e.g., Brennen et al., 2021), our results bring new insights into the persuasiveness techniques of such messages. In particular, we find that non-verbal displays are more likely to be associated with the attribution of blame for the problems identified in the posts, and that these associations vary with the target of the blame. The play on inappropriate displays, connecting powerful individuals (be it politicians or Bill Gates) with positive facial emotional displays in the face of people’s misery, is one regularity that emerges from our data analysis; the use of negative displays in connection with blaming group actors, such as the government or private companies, is another. As we elaborate in our concluding discussion, these framing choices are bound to arouse strong negative emotions in viewers and may enhance the power of disinformation messages—as well as make the debunking harder.

In what follows, we briefly discuss previous directions and work in persuasive framing, as well as in delineating the unique role played by human faces and non-verbal displays in encapsulating information that is being subconsciously decoded in mere tens or hundreds of milliseconds. We then discuss previous work on visuals in disinformation and introduce our four research questions. The third section describes the data and the methodological approach. The fourth presents the results of our analysis. The fifth section concludes.

## Previous Research

### Framing

As a large body of research demonstrates, framing is an intrinsic component of communication, and a powerful tool in enticing the targets of communication to agree with a message [see reviews by Chong and Druckman (2007a) and Scheufele (1999)]. This persuasive effect is particularly consequential when the frame is not rebuked, as evidenced by experimental research (e.g., Nelson et al., 1997; Chong and Druckman, 2007b). This is worth keeping in mind in the context of the present research, as despite the time and effort invested by fact-checking agencies to debunk fake claims (i.e., offer a counter-frame), it is not clear how many of those exposed to disinformation are also exposed to these corrections; and even when corrections are present, the effect of the initial claims may still persist (e.g., Thorson, 2016).

Notwithstanding the developments over the decades in the study of framing, a widely used approach makes use of the definition of framing proposed by Entman about 30 years ago as “[selecting] *some aspects of a perceived reality* and make them more salient in a communicating text, in such a way as to promote a particular *problem* definition, *causal interpretation*, moral *evaluation*, and/or treatment *recommendation*” (Entman, 1993, p.52, emphasis added). More than other theoretical frameworks—that offer more finely defined approaches to framing [e.g., the distinction between generic and issue-specific frames, e.g., see De Vreese (2005), or the distinction between episodic and thematic frames, as in Iyengar (1991)]—Entman’s minimalistic definition allows us to better explore the architecture of the disinformation claims, by virtue of imposing very few constraints on the content of the “problem,” (what is presented as being of concern) “causal interpretation” (who or what is responsible for the problem) “evaluation” (what motivates those to blame) or “recommendation” (the solution, if any proposed). We therefore adopt these categories in the coding of the posts, as described in the next section.

### The Role of Visuals in Framing

Visual elements have long been recognized as a key framing device (e.g., Gamson and Modigliani, 1989; Coleman, 2010). Owing to an evolutionary development over a much longer time span than the ability to process verbal content, the human brain processes visuals much quicker, before individuals are even aware of their presence (e.g., Grabe and Bucy, 2009).

Among the various visual categories, non-verbal displays are decoded particularly fast. Human faces are automatically processed less than fifty milliseconds after exposure (Grabe and Bucy, 2009, p. 13–14), and are interpreted as a rich source of information about the individuals in question [e.g., Willis and Todorov (2006); see also review by Dumitrescu (2016)]. Humans are, moreover, also very quick at consciously identifying positive facial emotions from negative ones, in less than half a second [see work by Bijlstra et al. (2010)]. Faces are not the only aspect that is automatically evaluated and integrated in judgments about the others—so is their body pose (e.g., Spezio et al., 2012). As Matsumoto et al. (2016) summarize in their overview of the literature, different body poses are associated with positive or negative attitudes and individual characteristics: “open arms and legs in a seated position generally communicate a more positive attitude and openness, whereas arms akimbo (arms on hips) or arms crossed in front of one’s body generally are associated with more negative attitudes. Open body postures also communicate more power” (2016, p. 387). Unsurprisingly—given the rich amount of information encapsulated in the human face and body—, facial displays, body postures and gestures are important conduits for the expression of emotions in multimodal communications (see, e.g., Poggi et al., 2013; Bucy et al., 2020), and play a key role in the connections leaders forge with their viewers and followers (e.g., Dumitrescu et al., 2015; Scardigno et al., 2021).

### Visuals and (COVID-19) Disinformation

Previous literature on disinformation presents experimental evidence that visuals can enhance the appeal of a disinformation message: Hameleers et al. (2020) find that multimodal disinformation is seen as more credible than simple textual information in one of the two topics they examine, related to refugees and terrorism. Directly in line with the aim of this study, Brennen et al. (2021) provide some valuable insights into the uses of visuals in COVID-19 disinformation claims. Their pioneering work in this direction finds that visuals’ roles in the construction of the disinformation message are often to, one, serve as direct evidence, and two, to illustrate and selectively emphasize aspects of the claim being put forth. A small percentage of them uses visuals to falsely impersonate authorities. Brennen et al.’s (2021) results draw attention to the ability of visuals to represent potentially abstract concepts and entities, and thereby to bring them into a more vivid existence, which in turn could make the disinformation claims more memorable [thereby echoing research by Vaccari and Chadwick (2020)].

The present study builds on this work to further inform the role of visuals in COVID-19 disinformation. In particular, Brennen et al. (2021) do not distinguish between specific visual elements. Their results are, therefore, unable to speak to the way in which features that viewers are bound to process automatically, such as non-verbal displays, contribute to the framing of the COVID disinformation claims. Moreover, the database used in the article ends in March 2020, before the first lockdowns came into full effect. Finally, whereas the geography of the disinformation posts plays little role in their data collection, we aim to further examine how such framing may vary by region within a well-defined geographic space—Europe.

In light of these aims, the present study poses the following *research questions:*

RQ1: How do *positive facial displays of emotion* interact with framing elements in the construction of COVID-19 disinformation messages in Europe in April-May 2020?

RQ2: How do *negative facial displays of emotion* interact with framing elements in the construction of COVID-19 disinformation messages in Europe in April-May 2020?

RQ3: How do *expansive body displays* interact with framing elements in the construction of COVID-19 disinformation messages in Europe in April-May 2020?

RQ4: How do *contractive body displays* interact with framing elements in the construction of COVID-19 disinformation messages in Europe in April-May 2020?

## Methodological Approach: Data and Coding

To address our research questions, we use the COVID-19 related disinformation messages indexed in the CoronaVirusFacts Database by the IFCN for the months of April 2020 and May 2020 in Europe. The IFCN is leading an alliance of “more than 100 fact-checkers around the world in publishing, sharing and translating facts surrounding the new coronavirus” Fighting the Infodemic (2021) who report to the IFCN disinformation messages they debunked in their country. The CoronaVirusFacts Alliance spans over 70 countries worldwide.

### Sample

In April and May 2020, the IFCN indexed 348 posts in the CoronaVirusFacts Database in Europe, from 18 countries—Belgium, Bosnia and Herzegovina, Croatia, the Czechia, Denmark, France, Georgia, Greece, Ireland, Italy, Latvia, Lithuania, Netherlands, Portugal, Spain, Serbia, Ukraine, and the United Kingdom. Of these entries, only 255 had working links to the original disinformation message (in addition to the link to the fact-check article). In this study, we analyze the original posts that also contained a visual component—either in the form of a static image or a video screen capture (*N* = 174). Table 1 presents the distribution of disinformation posts by country and month, and gives an overview of the number of images by post.

**TABLE 1:** Descriptive information about the disinformation messages in the sample, by country, month, and number of images in the post.

Country	Region	Frequency	Percent
United Kingdom	Western Europe	13	7.47
Ireland	Western Europe	9	5.17
Belgium	Western Europe	5	2.87
Netherlands	Western Europe	5	2.87
France	Western Europe	1	0.57
Italy	Southern Europe	38	21.84
Portugal	Southern Europe	20	11.49
Spain	Southern Europe	16	9.2
Greece	Southern Europe	4	2.3
Croatia	Central-Eastern Europe	24	13.79
Bosnia and Herzegovina	Central-Eastern Europe	18	10.34
Georgia	Central-Eastern Europe	3	1.72
Ukraine	Central-Eastern Europe	2	1.15
Lithuania	Northern Europe	8	4.6
Denmark	Northern Europe	7	4.02
Latvia	Northern Europe	1	0.57
Total		174	100

**Month**		**Frequency**	**Percent**
April 2020		94	54.02
May 2020		80	45.98
Total		174	100

**Number of pictures/post**		**Frequency**	**Percent**
Posts with 1 picture		139	79.89
Posts with 2 pictures		16	9.20
Posts with 3 pictures		6	3.45
Posts with 4 pictures		9	5.17
Posts with 5 pictures		4	2.30

Total number of pictures		245	

### Coding Scheme

All the posts were analyzed using a coding scheme that, in line with Entman’s (1993) definition of framing, included multiple variables for *aspect of reality—i.e., topic*, *problem* identified, *causal interpretation* focused on who or what is to be *blamed for the problem*, evaluation for *why* this is happening (the reasons for the behavior of those to be blamed), and *solution*. In addition, we also coded for the presence of a variety of specific visual and verbal devices, as well as for whether the frame elements were expressed visually and/or verbally. The coding scheme (included in full in the Supplementary Appendix) comprised 93 variables coded at the disinformation post level, and 37 variables coded at the image level (thus, the total number of variables coded ranged from 131 for messages containing just one image to 283 variables for messages containing five images).

Under the umbrella of each major framing component, we included several dichotomous variables that identified the presence of specific elements. For example, under the umbrella of *“topic”* ten different dichotomous yes-no variables helped further specify the topic as related to “vaccine,” “masks,” “lockdown,” “social distancing,” “disease contagion/spread/extent,” “disease consequences,” “disease cure,” “testing,” “technology-related,” and “economy-related.” An eleventh sub-variable identified the presence of any other topic not included in the list. In the category of *“problem,”* eight yes-no variables further specified its nature as “death,” “illness,” “dehumanizing,” “freedom of movement,” “freedom of speech/expression,” “truth,” “Big Brother control,” or “financial/economic loss.” A further ninth variable indexed any other problem not listed above. Under the umbrella of *“blame,”* related to the causal interpretation, two main groups of dichotomous variables first indicated whether the blame (if mentioned) was assigned to an “identified individual,” “identified group/institution/organization,” or some “other category.” In this case, the coders also indicated whether the target of the blame was a “politician,” a “businessperson,” a “scientist,” “the government,” a “non-governmental national authority,” an “International Organization,” a “private company,” a “party,” an “ethnic, sexual, political, or religious minority,” or “scientists as a community.” Two other variables indexed the presence of any other individual or group not mentioned above. We had initially also included several specific variables for the evaluation of *“why”* the problem is happening and for *“solution,”* however, these were dropped from the coding scheme after coding about a quarter of the posts, since they were found to be rarely present. Finally, the coders also recorded whether, taking every aspect of the message into account, the main topic, the main problem, or the blame target were *expressed “visually”* or *“verbally.*”

The *image-level variables* covered a wide variety of features. We documented whether each image showed any individual, and how many, as well as whether that (those) individual(s) were “human exemplars,” “politicians,” “individual experts,” “contested experts” (i.e., experts presented in this capacity in the message, but not widely accepted as such), or any other “well-known figure” (such as businesspeople, cultural elites etc.). We also coded for any visual representation of institutions (though such signs as flags or logos), for any medical personnel or equipment, for any non-medical technological equipment, as well as for any pop-culture elements.

Among the “person” variables, we coded for the presence of faces (fully or partially visible), eye-contact, as well as four *non-verbal displays of interest: facial expressions and body poses* (variables #116–126 in the coding scheme). The valence of the facial emotional displays was first recorded as “positive” or “negative.” Further variables helped refine whether the positive emotional display was indicative of “happiness,” or whether the negative emotional display was indicative of “anger,” “anxiety,” “disgust,” or “other negative emotion.” Finally, body pose was coded as “expansive” or “contractive.”

### Coding Training and Instructions

The coding scheme was discussed at length prior to the start of the coding, and annotated heavily with more examples and more detailed instructions after the double-coding of the first 20 posts and then again after the double coding of the second batch of 20 posts. The coders were instructed to include a topic if key words or representative visuals were present (for example, to code for “topic is vaccine” if the word “vaccine” was mentioned or if the post included a picture of a syringe). Coders were also instructed to choose yes for the visual representation of a topic, problem, or blame if those elements were reflected in the visuals (e.g., the depiction of masks would count as a visual representation of the topic of “masks,” the depiction of Bill Gates would count as a visual representation of “blame,” the depiction of people on hospital beds would count as a visual representation of the problem being “illness”). Each post was coded in an excel file sheet, which contained the instructions to the left of each variable. For each variable, the coders could choose yes or no (with one exception, the variable asking how many individuals were depicted, where the coders had to count). Additionally, when politicians, businesspeople or experts where visible or mentioned, coders specified who was represented. The variables were not mutually exclusive, for example, a message could contain more than one topic if the key words were present, and the same principle applied for problems or blame attribution.

### Visual Coding

Facial emotions were coded following Ekman and Friesen’s (2003) description of the facial muscles’ positions associated to displays. Coders were explicitly instructed to follow Ekman and Friesen’s (2003) account when reporting both the overall valence of the display (positive or negative), and specific emotions (happiness, fear, anger, and disgust); they were instructed to select “no” if they were not sure of the valence or of the particular emotion.

In line with Matsumoto et al. (2016), body posture was assessed by examining the position of an individual’s limbs with respect to their body. In this respect, we followed Wasike’s (2019) instructions, and coded as “expansive body pose” when an individual “[took] more space or [had] limbs extended from the body (e.g., raised arms, hands gesticulating away from the body, or legs spread when seating or standing)” (Wasike, 2019, p. 258) and as “contractive body pose” when an individual “[kept] limbs close to the body” (Wasike, 2019, p.258). Coders were instructed to code “yes” for a particular body pose only when certain, and choose “no” otherwise.

Finally, coders were instructed to code “human exemplars” as “yes” if the post depicted ordinary people, but not people shown to symbolize a particular job (such as nurses or doctors, these were counted as medical personnel; or soldiers, these were counted as others).

### Disinformation Post Languages and Translation Policy

The posts were in the original language corresponding to the country of dissemination, namely Bosnian, Croatian, Danish, Dutch, English, French, Georgian, Greek, Italian, Lithuanian, Portuguese, Russian, Spanish, and Ukrainian. They were coded by the two authors, who had both fluent knowledge of English and, in between them, fluent knowledge of French and Italian, intermediary knowledge of Spanish, and native knowledge of Serbian. All the non-English posts were first translated into English with Google Translate. Google Translate uses a Recurrent Neural Network (RNN)-based model called Neural-Network for Machine Translation, introduced in 2016 (Le and Schuster, 2016), and perfected in 2020 (Caswell and Liang, 2020). While comprehensive tests of its accuracy are hard to get by, at least two recent papers in the medical and the hospitality domain suggest this is quite high. Taira et al. (2021) looked at the accuracy of translations of commonly used hospital discharge instructions English into Spanish, Chinese, Vietnamese, Tagalog, Korean, Armenian, and Farsi. They found that the overall meaning was preserved in 85.5% of their sample (with Spanish having the highest accuracy translations at 94%). Conversely, Hasyim et al. (2021), examined the accuracy of culinary recipes translations from French into Indonesian, and reported similarly high results.^1^

In addition, given the diversity of countries from which the sample originated, it was unavoidable that some posts would depict individuals the coders would be unfamiliar with. In that case, in order to address some of the intercultural differences that inevitably arise with such a diverse sample, Google Image was used to reverse-search the unknown individuals’ identity and obtain a detailed account of their profile, to guarantee the coding accuracy and cross-country reliability.

### Validation Approach

About 40% of the sample (70 disinformation messages) was double coded. For the first 40 posts, coders discussed each difference and agreed on a common solution. For the remaining 30 posts we calculated the percentage agreement—which stood at 94%.

## Results

We begin with section “Descriptive Results” by looking at the distribution of frame components and how they are communicated over the two months and the four European regions covered in our data (as the number of posts per country is too small in some cases to warrant a country-based analysis). We describe the significant patterns in the text and relegate all the graphs in the Supplementary Figures 1–8. Then, in section “Non-verbal Displays and Framing Components,” we examine how the four non-verbal displays—positive facial emotional displays, negative facial emotional displays, contractive body poses, and expansive body poses—vary with the topics, problems, and blame attribution targets. The Section “Non-verbal Displays and the Visual Expression of Framing Components” presents the association between non-verbal displays and the visual expression of framing components. In the Section “Type of Individuals Depicted and Non-verbal Displays,” we examine how different individual types are associated with these four non-verbal displays. Finally, in the Section “Discussion,” we summarize our main results and discuss their significance.

### Descriptive Results

#### Framing Components Across Time and Region

##### Topic Coverage

Simple chi-square tests show that the topic of vaccination and lockdown marginally picked up in the month of May 2020, in comparison to April (Vaccination chi-square = 3.469, *p* = 0.063, Lockdown chi-square = 2.799, *p* = 0.094), while the topic of disease contagion and extent became significantly less frequently (Contagion chi-square = 7.550, *p* = 0.006).

As can be seen in Supplementary Figure 1, we observe some variation in the topic presence across the geographic space. In Northern Europe (counting Denmark, Lithuania, and Latvia), 90% of the posts featured references to disease contagion, a significantly higher percentage than the range of 49–60% in the other regions (chi-square = 10.730, *p* = 0.013). The Northern European posts also featured disease consequences more heavily (in about 70% of the posts, compared with about 30% in each of the other regions, chi-square = 10.231, *p* = 0.017). In addition, a significant difference emerges in the use of adjacent topics, such as high-level technology (5G) and economic worries. None of these topics are covered in Western European posts, and there is limited coverage in Southern Europe; conversely, 5G is covered in about a quarter of the posts in Central-Eastern Europe, and in about a fifth of the posts in Northern Europe. A similar distribution is observed for economic worries (as displayed in Supplementary Figure 1).

##### Problems Identified

As Supplementary Figure 2 shows, the curtailing of freedom of movement features more frequently in the May posts (chi-square = 4.143, *p* = 0.042), but all the other problems surface at the same rate in both months.

The geographic distribution reveals a slightly different disinformation diet in Central-Eastern and Northern Europe compared to Western and Southern Europe. First, there are significantly more frequent references to the nefarious effects of technological advances on humans (5G mainly) in Central and Eastern Europe than in Western and Southern Europe (chi-square = 17.865, *p* = 0.000). “Big Brother control” is identified as a problem by 40% of the Central-Eastern European posts and 31% of the Northern European ones, compared to 19% of the Southern European and only 6% of the Western European ones (chi-square = 14.3705, *p* = 0.002). Although it appears more rarely as a problem than others, economic loss is more prominent in Central-Eastern and Northern Europe than in the rest of Europe (chi-square = 8.719, *p* = 0.033).

##### Blame Attribution

There is an increase in the frequency of blame being at least partly attributed to a group (be it organization or institution) from 1 month to the other, with about 60% of the posts doing so in May, compared to only about 20% of the posts in April (chi-square = 24.065, *p* = 0.000, see Supplementary Figure 3). Conversely, we observe a decrease in the attribution of blame to non-human agents (e.g., the virus itself), with only 10% doing so in May compared to about 30% in April (chi-square = 8.870, *p* = 0.003).

In terms of geographic distribution, the posts in Northern Europe are significantly more likely to identify non-human agents to blame (with about 44% of the posts doing so, chi-square = 8.994, *p* = 0.029).

##### Specific Targets in Blame Attribution

Businesspeople (especially Bill Gates), the government, private companies (such as Microsoft or social media providers), political parties and the mass media featured more heavily among the blame targets in May than in April (the lowest chi-square = 3.864, *p* = 0.049).

When looking at European regions, there is little differentiation by blame target, except for businesspeople, who featured more heavily in posts in Central-Eastern Europe and Northern Europe than in the rest of Europe (chi-square = 8.222, *p* = 0.042, see Supplementary Figure 4).

#### How Are Frames Communicated?

We next turn to simple descriptive information about the use of verbal and non-verbal elements in the 174 posts, as they vary across time and space in our sample.

##### Verbal and Non-verbal Means of Conveying Frame Elements

The variables registering the use of verbal and visual ways of expression were coded at the level of the post, for each frame component. There is limited difference by month in the use of verbal means to directly express the topic or the problem. However, verbal means became more prevalent in the attribution of blame in May (chi-square = 4.525, *p* = 0.033). Region-wise, there are also limited differences in the use of verbal communication in framing, with a similar exception in the expression of the attribution of blame. The percentage of posts using verbal means for this purpose is significantly lower in Western Europe (at 45%) than elsewhere on the continent, and especially in comparison to Central-Eastern Europe, where verbal attribution of blame is present in 77% of the posts (chi-square = 8.328, *p* = 0.040, see Supplementary Figure 5).

The use of non-verbal means to express the various frame components varied also relatively little across the time period, but there appears to be a trend toward lower reliance on visuals to express the topic (chi-square = 9.320, *p* = 0.00, see Supplementary Figure 6). At a regional level, disinformation posts in Western Europe are more likely than in the other regions to rely on non-verbal representations of the topic (with 88% of the posts using this communication channel); at the lower end, the proportion of posts expressing the topic visually is only at 60% in Central-Eastern Europe (chi-square = 8.850, *p* = 0.031).

##### Who Is Featured?

Image-specific features such as who is pictured, facial emotional displays and body poses were coded at the image level (*N* = 245). The use of individuals in images registers a significant increase from April to May (chi-square = 4.815, *p* = 0.028), but there is limited change in who is pictured across time. On a regional basis, as can be also seen from Supplementary Figure 7, images of experts tend to feature slightly more frequently in Southern Europe (chi-square = 6.552, *p* = 0.088), and images of well-known other elites (such as Bill Gates) appear to be marginally more present in Central-Eastern Europe (chi-square = 6.976, *p* = 0.073).

##### Emotional Displays and Body Poses

The distribution of emotional displays and body poses in the 245 images is displayed in Supplementary Figure 8. As can be seen, there is no significant change in the frequency of these non-verbal displays across the time period under study or between the various European regions.

### Non-verbal Displays and Framing Components

We next examine how framing components (such as topic, problems, and attribution of responsibility) are associated with the choice of a non-verbal display in an image. The analyses all follow the same pattern. In each model the dependent variable is dichotomous and represents whether the image features or not a particular facial or body pose display. We run models with four different non-verbal displays as dependent variables: positive emotional display, negative emotional display, expansive body pose and contractive body pose. The independent variables represent either the topics, problems and blame elements identified in the posts (coded at the level of the post) or the choice of visual elements (the type of individual featured, coded at the level of the image). Since the non-verbal displays are coded at the image level, the analyses are run on the entire sample of images (*N* = 245) with bootstrapped corrected and accelerated (BCA) standard errors, clustered by disinformation post (*N* = 174 clusters).

#### Non-verbal Displays and Topics in Disinformation Messages

As can be seen in Model 1 in Table 2, the topic of vaccination is significantly associated with positive facial displays [the estimated marginal effect of the topic of vaccination on the probability of having a positive emotional display is 0.25, *p* = 0.000, Bootstrap 95% CI = (0.113, 0.384)], but the model fit for all the topics is poor [pseudo-Rsq = 0.08, Wald chi(11) = 16.83, *p* = 0.11]. Model 2 displays the impact of the choice of topic on the likelihood of a contractive body pose. Two topics stand out: disease contagion, which is associated with an increase in the display of contractive body poses [with an estimated marginal effect of 0.162, *p* = 0.026, Bootstrap 95% CI = (0.019, 0.311)], and disease cure, which makes contractive body poses significantly less likely [with an estimated marginal effect of −0.275, *p* = 0.000, Bootstrap 95% CI = (−0.415, −0.135)]. There are no further differences between different topics in their association with negative facial displays (pseudo-Rsq = 0.02, Wald chi = 3.98, *p* = 0.97) or with expansive body poses (pseudo-Rsq = 0.04, Wald chi = 7.21, *p* = 0.78).

**TABLE 2:** Non-verbal displays and topics in disinformation messages.

	Model 1. Dependent Variable: Positive Facial Emotional Display (Yes/No)					Model 2. Dependent Variable: Contractive Body Pose (Yes/No)				
	Observed Coef.	Bias	Bootstrap Std. Err.	[95% BCA Conf. Interval]		Observed Coef.	Bias	Bootstrap Std. Err.	[95% BCA Conf. Interval]	
Topic: Vaccine	1.543	0.091	0.459	0.597	2.362	0.906	0.109	0.564	−0.259	1.902
Topic: Masks	−0.261	−0.001	0.604	−1.451	0.895	−0.178	0.060	0.730	−1.608	1.285
Topic: Lockdown	−0.527	0.021	0.542	−1.653	0.480	−0.347	−0.120	0.570	−1.323	0.924
Topic: Social distancing	0.657	0.007	0.669	−0.708	1.922	−0.392	−0.046	0.901	−1.859	1.840
Topic: Disease contagion/spread/extent	0.401	0.038	0.417	−0.349	1.279	1.425	0.155	0.672	0.171	2.642
Topic: Disease consequences	−0.468	0.041	0.388	−1.266	0.316	0.016	−0.029	0.469	−0.851	0.851
Topic: Disease cure	0.157	0.137	0.538	−1.023	1.097	−2.368	0.149	0.518	−3.791	−1.580
Topic: Testing	−0.294	−0.097	0.861	−1.951	1.227	0.603	−0.113	0.736	−1.011	2.007
Topic: Technology-related	−0.989	−0.176	0.725	−2.417	0.234	−1.536	−0.012	0.958	−3.519	0.301
Topic: Economy-related	0.710	0.012	0.582	−0.474	1.854	0.824	−0.094	0.627	−0.427	2.098
Topic: Other category	0.474	0.040	0.437	−0.479	1.263	0.288	−0.061	0.573	−0.747	1.420
Constant	−1.706	−0.149	0.429	−2.389	−0.870	−2.772	−0.171	0.660	−3.929	−1.654
N		245				245				
Replications		1416				755				
Wald chi2 (9)		16.83				26.83				
Prob > chi2		0.113				0.005				
Pseudo R2		0.077				0.115				
Log pseudolikelihood		−121.56				−92.054				
Replications based on 174 clusters in id										

	**Marginal Effects on P (Positive Facial Emotional Display)**					**Marginal Effects on P (Contractive Body Pose)**				
		
	**Average marginal effect**	**Bootstrap Std. Err.**	**P>| z|**	**[95% Conf. Interval]**		**Average marginal effect**	**Bootstrap Std. Err.**	**P>| z|**	**[95% Conf. Interval]**	

Topic: Vaccine	0.248	0.069	0.000	0.113	0.384	0.105	0.063	0.098	−0.019	0.229
Topic: Masks	−0.042	0.097	0.666	−0.232	0.148	−0.021	0.085	0.808	−0.187	0.146
Topic: Lockdown	−0.085	0.087	0.330	−0.255	0.086	−0.040	0.066	0.542	−0.169	0.089
Topic: Social distancing	0.106	0.107	0.321	−0.103	0.315	−0.045	0.105	0.664	−0.251	0.160
Topic: Disease contagion/spread/extent	0.065	0.067	0.334	−0.066	0.196	0.165	0.074	0.026	0.020	0.311
Topic: Disease consequences	−0.075	0.063	0.228	−0.198	0.047	0.002	0.054	0.974	−0.105	0.108
Topic: Disease cure	0.025	0.086	0.769	−0.144	0.194	−0.275	0.071	0.000	−0.415	−0.135
Topic: Testing	−0.047	0.138	0.732	−0.318	0.223	0.070	0.086	0.414	−0.098	0.238
Topic: Technology-related	−0.159	0.115	0.167	−0.385	0.067	−0.178	0.109	0.104	−0.393	0.036
Topic: Economy-related	0.114	0.092	0.214	−0.066	0.294	0.096	0.071	0.181	−0.044	0.236
Topic: Other category	0.076	0.070	0.275	−0.061	0.213	0.033	0.066	0.616	−0.097	0.164

#### Non-verbal Displays and Problems Identified by Disinformation Messages

As Model 3 in Table 3 shows, the presence of non-verbal displays is not, in our sample, significantly associated with the specific problems identified in the post, with one potential exception. The average marginal effect of “death” on the likelihood of positive emotional display is estimated at 0.123, but does not reach the level of conventional significance (*p* = 0.083).

**TABLE 3:** Positive facial emotional displays and problems in disinformation messages.

	Model 3. Dependent Variable: Positive Facial Emotional Display (Yes/No)				
	Observed Coef.	Bias	Bootstrap Std. Err.	[95% BCA Conf. Interval]	
Problem: Death	0.725	0.099	0.432	−0.140	1.471
Problem: Illness	−0.383	0.048	0.422	−1.302	0.387
Problem: Dehumanizing	0.233	−0.083	0.687	−1.136	1.519
Problem: Freedom of movement	0.116	0.077	0.535	−1.318	0.963
Problem: Freedom of speech/expression	−0.213	−0.022	0.835	−1.871	1.506
Problem: Truth	0.425	0.063	0.398	−0.423	1.118
Problem: Big Brother control	0.339	0.011	0.655	−1.130	1.521
Problem: Financial/Economic loss	−0.005	−0.147	0.769	−1.394	1.617
Problem: Other	0.083	−0.050	0.622	−1.186	1.288
Constant	−1.624	−0.164	0.435	−2.326	−0.727
Number of obs		245			
Replications		1486			
Wald chi2 (9)		5.60			
Prob > chi2		0.779			
Pseudo R2		0.034			
Log pseudolikelihood		−127.251			
Replications based on 174 clusters in id					

	**Marginal Effects on P (Positive Facial Emotional Display)**				
	
	**Average marginal effect**	**Bootstrap Std. Err.**	**P>| z|**	**[95% Conf. Interval]**	

Problem: Death	0.123	0.071	0.083	−0.016	0.262
Problem: Illness	−0.065	0.071	0.362	−0.205	0.075
Problem: Dehumanizing	0.040	0.116	0.733	−0.187	0.266
Problem: Freedom of movement	0.020	0.091	0.829	−0.158	0.198
Problem: Freedom of speech/expression	−0.036	0.143	0.800	−0.316	0.243
Problem: Truth	0.072	0.067	0.283	−0.060	0.204
Problem: Big Brother control	0.058	0.112	0.608	−0.162	0.277
Problem: Financial/Economic loss	−0.001	0.130	0.994	−0.257	0.255
Problem: Other	0.014	0.106	0.894	−0.193	0.221

#### Blame Attribution and Non-verbal Displays

**FIGURE 1:**
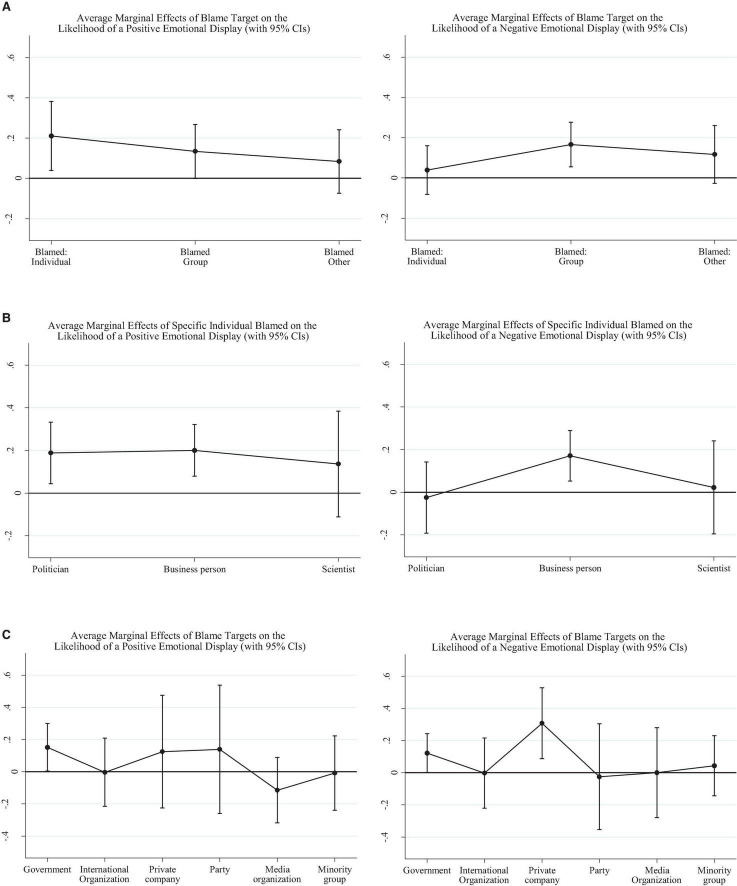
**(A)** The average marginal effects of generic blame targets on the likelihood of a positive emotional display (left-side graph) and negative emotional display (right-side graph). **(B)** The average marginal effects of specific individual blame targets on the probability of a positive facial emotional display (left) and negative facial emotional display (right). **(C)** The average marginal effects of specific group blame targets on the probability of a positive facial emotional display (left) and negative facial emotional display (right).

More so than the topic or the problems identified in the posts, blame attribution is significantly associated with positive and negative emotional displays, as can be seen in Figures 1A–C. The corresponding tables are presented in the Supplementary Tables 1A–C. There are no significant associations between blame attribution and body poses.

The left graph in Figure 1A presents the average marginal effects of the generic blame target in the post on the likelihood of a positive emotional display. Assigning blame to a specific individual (as opposed to a group, to a non-human agent, or to no blame at all) is associated with an increase in the likelihood of a positive facial emotional display of 0.21 [*p* = 0.016, Bootstrap CI = (0.039, 0.382)]. The average marginal effect of assigning blame to a group (e.g., whether to an institution, a party, a company, or a minority) almost reaches significance too, being estimated at 0.14 [*p* = 0.051, Bootstrap CI = (−0.001, 0.268)]. Conversely, as can be seen in the right-side panel of Figure 1A, having a group blame target is significantly associated with a higher likelihood of a negative emotional display, with an average marginal effect of 0.17 [*p* = 0.003, Bootstrap CI = (0.055, 0.276)].

When we look into more detail at the specific individuals blamed, as in Figure 1B, we see that politicians and businesspeople are both more likely to be paired with a positive facial emotional display; a blamed politician is associated, on average, with a 0.19 increase in the probability of a positive display [*p* = 0.011, Bootstrap CI = (0.044, 0.333)], while a blamed businessperson leads to an estimated increase of 0.20 in the same probability [*p* = 0.000, Bootstrap CI = (0.079, 0.322)]. Negative displays, as can be seen in the right-side graph of the Figure 1B, are associated with businesspeople; when businesspeople are blamed, the likelihood of a negative emotional display increases, on average, by 0.17 [*p* = 0.005, Bootstrap CI = (0.052, 0.289)].

Finally, the influence of the specific group blamed on the likelihood of emotional displays is graphed in Figure 1C. Blaming the government is associated with increases in both positive and negative emotional displays: in this case, the likelihood of the former is estimated to increase on average by 0.15 [*p* = 0.044, Bootstrap CI = (0.004, 0.300), left-side graph], while the likelihood of the latter is estimated to be on average 0.12 higher [*p* = 0.046, Bootstrap CI = (0.002, 0.243), right-side graph]. Furthermore, the likelihood of a negative display also goes up by about 0.31 [*p* = 0.006, Bootstrap CI = (0.087, 0.528)] when private companies [such as Microsoft, or Facebook or YouTube (or Google, implicitly)] are to blame.

### Non-verbal Displays and the Visual Expression of Framing Components

**FIGURE 2:**
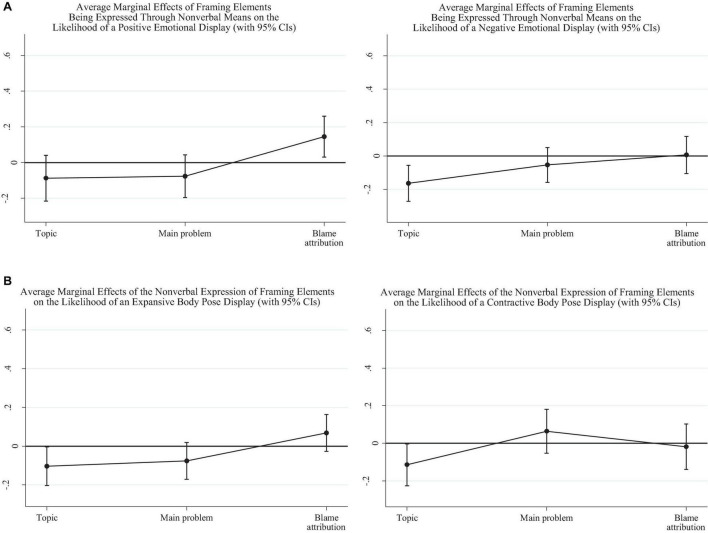
**(A)** Average marginal effects of the non-verbal expression of framing elements on the likelihood of a positive facial emotional display (left-side graph) and negative facial emotional display (right-side graph). **(B)** Average marginal effects of the non-verbal expression of framing elements on the likelihood of an expansive body pose (left) and contractive body pose (right).

We next look at whether the non-verbal expression of the framing elements is itself associated with specific non-verbal displays. Figure 2A presents the estimated marginal effects of the “topic,” “problem,” and “blame attribution” being expressed in the disinformation post through non-verbal means on the likelihood of the images associated with the post displaying either positive or negative facial emotion. The corresponding tables of full results is presented in the Supplementary Tables 2A,B).

As we see in the left-side graph of Figure 2A, when blame is expressed through non-verbal channels, displays of positive facial emotions are significantly more likely, by about 0.15 [*p* = 0.013, Bootstrap CI = (0.031, 0.260)]. Conversely, the only significant effect in the right-side graph of Figure 2A is the one for the non-verbal expression of the main topic. In fact, when the topic is expressed through non-verbal means, it is less likely for any image in the post to also include a negative facial display, by about −0.16 [*p* = 0.003, Bootstrap CI = (−0.271, −0.055)].

Figure 2B presents the impact of the non-verbal expression of framing elements on the likelihood of an expansive or contractive body pose. The two graphs show that the non-verbal expression of the main topic in the disinformation post is associated to a significant decrease in the probability of having either body pose type by about −0.11.

### Type of Individuals Depicted and Non-verbal Displays

**FIGURE 3:**
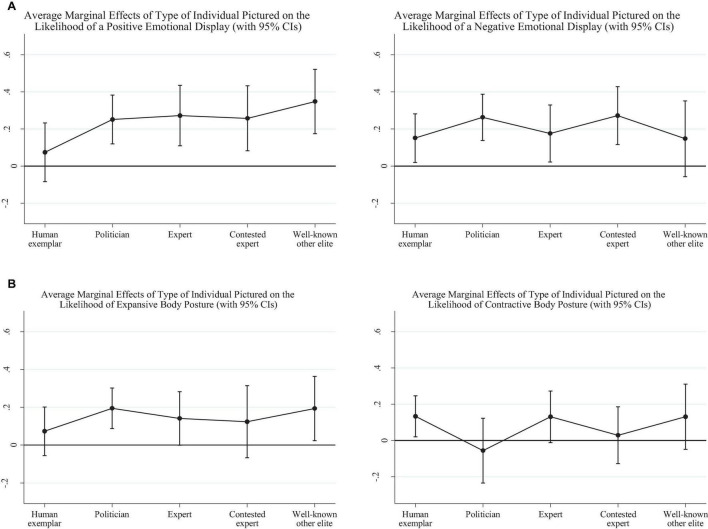
**(A)** Average marginal effects of the type of individual pictured on the likelihood of a positive facial emotional display (left-side graph) and negative facial emotional display (right-side graph). **(B)** Average marginal effects of the type of individual pictured on the likelihood of an expansive body pose display (left) and a contractive body pose display (right).

Finally, we look at the association between specific individual types pictured and the likelihood of having any of the four non-verbal displays in each image. Figure 3A presents the influence of picturing a human exemplar, a politician, an expert, a contested expert, or a well-known other elite, on the likelihood of a positive or negative facial emotional display. Figure 3B presents the same results for the probability of an expansive or contractive body pose. Supplementary Tables 3A,B present the full results.

As illustrated in Figure 3A, human exemplars and well-known elites are associated with opposite patterns of facial displays. Where depictions well-known elites (such as Bill Gates) are linked to an increase in the likelihood of positive facial emotion [of 0.35, *p* = 0.000, Bootstrap CI = (0.174, 0.521)], human exemplars are linked, in reverse, to an increase in negative emotional displays [by 0.15, *p* = 0.024, Bootstrap CI = (0.020, 0.281)]. Apart from well-known elites, who are likely to be only pictured with a smile on their face, all other individuals holding public status are associated with increases in both positive and negative facial emotional expressions.

Figure 3B depicts the impact of portraying different individual types on the likelihood of having an expansive (left-side graph) and contractive body pose display (right-side panel). Politicians and well-known elites are both more likely to sport an expansive body pose (with a similar increase in the likelihood of an expansive display of 0.19, both significant at *p* = 0.05). Experts also tend to be more associated with an expansive body pose, but the average marginal effect of 0.14 fails to reach significance (*p* = 0.052). Conversely, human exemplars are the only ones to be more likely to be associated with contractive body poses [with an average marginal effect of 0.13, *p* = 0.021, Bootstrap CI = (0.020, 0.246)].

### Discussion

Our analysis shows not only that the topics, problems identified, and blame targets in disinformation messages vary by European region, and from month to month (as in section “Descriptive Results”), but more importantly, that non-verbal displays play a significant role in how these messages are crafted.

An interesting result is that facial positive emotional displays are, perhaps unexpectedly, associated with vaccines, and with blaming well-identified individuals with decision-making power, such as politicians and businesspeople; they are also weakly associated with mentions of “death” as a problem—all three framing components carrying a strongly negative charge. When we look at the group targets identified for blame, the government is—similarly to politicians and businesspeople—also associated with the display of positive facial emotions. In fact, when blame is expressed visually, facial displays of positive emotion are likely to be present.

At the same time, blame attribution is also linked to negative displays. Thus, displays of facial negative emotion are more likely when businesspeople or the government are held for responsible; but also when private companies, as well as groups in general are to blame.

Overall, these results point not just to the strategic use of non-verbal elements in connection to blame attribution, but also to their likely role in increasing the negative emotional charge of the post. Previous work shows that inappropriate emotional displays (Bucy, 2000; Bucy and Bradley, 2004) elicit intense negative emotional reactions. Thus, in the context of disinformation posts non-verbal displays may contribute to arousing negative emotion both directly (when showing negative emotions) and indirectly (when inappropriately showing positive emotions).

Finally, our analyses also point to the use of non-verbal displays being implemented to mark a clear difference between the human exemplars, representing ordinary citizens, and those in power, whether political or financial. Human exemplars are more likely to be sporting a contractive body pose, suggesting a defensive position, while politicians and well-known elites are more likely to sport an expansive body pose, suggesting self-assuredness. Similarly, human exemplars are unlikely to smile, unlike individuals in power.

## Conclusion and Further Research Directions

Taken together, our results bring some important new insights into the architecture of disinformation claims and the roles of visuals in their construction. Previous literature has found that visuals add credibility to disinformation claims (e.g., Hameleers et al., 2020), and that in the context of COVID-19 disinformation, the visual side of the message serves primarily as evidence or to emphasize aspects of the claims (Brennen et al., 2021). To this, we add a new dimension: the likely role of non-verbal displays in ramping up negative emotions. The automatic processing of non-verbal displays, and their rich emotional and informational content [e.g., see review by Dumitrescu (2016)] make them in fact prime candidates for this role. Given that inappropriate displays arouse negative emotion (cf., e.g., Bucy, 2000), by pairing two unlikely extremes—strong positive emotion, expressed through non-verbal displays, and strongly negative verbal claims—disinformation creators can potentially heighten to the extreme the intensity of this emotional reaction. The specific emotion felt, whether fear, anger, disgust, or another, remains to be determined, and may in fact be dependent on the context. Previous crisis-related research (e.g., Wagner, 2014) indicates that strong anger rather than fear may prevail when smiling businesspeople or politicians are verbally blamed for the problems associated with Covid-19, but that is an empirical matter which should be further investigated.

Irrespective of the type of negative emotion, by heightening its intensity, disinformation architects also protect themselves from effective debunking, or at least, make debunking harder. In fact, inducing negative emotions, such as anxiety and anger, has been shown in turn to encourage holding less accurate beliefs when exposed to misinformation (e.g., Weeks, 2015). Anxiety acts to narrow the focus of attention to the immediate threat, and to bias the information search (e.g., Gadarian and Albertson, 2014); anger on the other hand, increases reliance on one’s predispositions and heightens perceptions of opposing information as biased (e.g., Suhay and Erisen, 2018). Either one of these emotions can, therefore, make citizens who have been exposed to disinformation oblivious to fact-checkers’ arguments.

While there is no easy solution to this problem, highlighting the emotional construction of the messages as part of the fact-check, as well as educating the public about the use of emotional extremes in the disinformation message construction (using positive non-verbal displays and negative “facts”), may help counter the effects of the emotional reactions on decision-making. This in turn, may make citizens more likely to consider fact-checkers’ arguments at their value.

The present analysis is limited, both in time, and place. Thus, the extent to which the observed patterns were maintained for the rest of the pandemic, and apply outside Europe is to be investigated. However, the results suggest that further research into the use of non-verbal displays in disinformation messages is acutely needed, as well as into the emotional activation strategies such messages depend on for their success.

## Data Availability Statement

The raw data supporting the conclusions of this article will be made available by the authors upon request, without undue reservation.

## Author Contributions

DD: data collection, data analysis, theoretical framework, and manuscript preparation. MT: data collection. Both authors contributed to the article and approved the submitted version.

## Conflict of Interest

The authors declare that the research was conducted in the absence of any commercial or financial relationships that could be construed as a potential conflict of interest.

## Publisher’s Note

All claims expressed in this article are solely those of the authors and do not necessarily represent those of their affiliated organizations, or those of the publisher, the editors and the reviewers. Any product that may be evaluated in this article, or claim that may be made by its manufacturer, is not guaranteed or endorsed by the publisher.

## Footnotes

1 While Google Translate still exhibits a certain amount of gender bias (e.g., Prates et al., 2020), and is not able to retain all the linguistic subtleties (Hofstadter, 2018), these features were not included in the coding scheme for this project, which was focused on key words and general meaning.
