# Supplementary material for: The Use of Non-verbal Displays in Framing COVID-19 Disinformation in Europe: An Exploratory Account
Source: Front Psychol. 2022 Mar 14;13:846250. doi: 10.3389/fpsyg.2022.846250 (PMC8964021; doi:10.3389/fpsyg.2022.846250)
Supplement: Supplementary file 1 [file Data_Sheet_1.pdf]

Appendix

Supplementary materials for
Dumitrescu, D. & Trpkovic, M. (2022) The Use of Non-verbal Displays in Framing COVID-19 Disinformation in Europe: An Exploratory Account. *Frontiers in Psychology*, 13,
doi: 10.3389/fpsyg.2022.846250

Contents

1. Coding Scheme 2

2. Framing Variables Distributions 16

Supplementary Figure 1. Proportion of Disinformation Messages by Topic (Left: by Month, Right: by Region) 16

Supplementary Figure 2. Proportion of Disinformation Messages Mentioning Specific Problems (Left: by Month, Right: by Region) 16

Supplementary Figure 3. Proportion of Disinformation Messages by Blame (Yes/No) and Generic Blame Targets (Left: by Month, Right: by Region) 17

Supplementary Figure 4. Proportion of Disinformation Messages with Specific Blame Targets (Left: by Month, Right: by Region) 17

Supplementary Figure 5. Proportion of Disinformation Messages using Verbal Means to Express Different Framing Elements (Left: by Month, Right: by Region) 18

Supplementary Figure 6. Proportion of Disinformation Messages Using Visual Means to Express Different Framing Elements (Left: by Month, Right: by Region) 18

Supplementary Figure 7. Proportion of Disinformation Messages Picturing Specific Individual Types (Left: by Month, Right: by Region) 19

Supplementary Figure 8. Proportion of Disinformation Messages Picturing Specific Emotional and Body Posture Displays (Left: by Month, Right: by Region) 19

3. Additional Tables 20

Supplementary Table A1. Nonverbal Displays and Generic Blame Targets 21

Supplementary Table B1. Nonverbal Displays and Individual Blame Targets 22

Supplementary Table C1. Nonverbal Displays and Group Blame Targets 23

Supplementary Table A2. Nonverbal Displays and Visual Expression of Framing Components 24

Supplementary Table B2. Nonverbal Displays and Visual Expression of Framing Components 25

Supplementary Table A3. Nonverbal Displays and Type of Individuals Depicted 26

Supplementary Table B3. Nonverbal Displays and Type of Individuals Depicted 27

1. Coding Scheme

order	Variable id	Variable label	Variable coding	**Note: code all the topics present in the post, <u>taking into account both the verbal and the visual elements</u>**	Comments
0.5	post id	Insert post id number	#######		
1	V1.1	1=TopicPRESENT: Vaccine	0=No/1=Yes	Yes if the post talks about vaccines, no matter the position taken	
2	V1.2	2=TopicPRESENT: Masks	0=No/1=Yes	Yes if the post talks about mask wearing, mask safety, or anything about marks, no matter the position taken	
3	V1.3	3=TopicPRESENT: Lockdown	0=No/1=Yes	Yes if the post talks about lockdown, no matter the position taken	
4	V1.4	4=TopicPRESENT: Social distancing	0=No/1=Yes	Yes if the post talks about social distancing, no matter the position taken	
5	V1.5	5=TopicPRESENT: Disease contagion/spread/extent	0=No/1=Yes	Yes if the post talks about how widespread covid is, such as by making reference to pandemic, or how easy/difficult it is to chatch it, no matter the position taken	
6	V1.6	6=TopicPRESENT: Disease consequences	0=No/1=Yes	Yes if the post talks about disease consequences on self, society, medical system, no matter the position taken	
7	V1.7	7=TopicPRESENT: Disease cure	0=No/1=Yes	Yes if the post advocates a cure	
8	V1.8	8=Topic PRESENT: Testing	0=No/1=Yes	Yes if the post is about tests or testing	

9	V1.9	9=TopicPRESENT: Technology-related	0=No/1=Yes	Yes if the post is about technology (e.g., 5G, chips)	
10	V1.10	10=TopicPRESENT: Economy-related	0=No/1=Yes	Yes if the posts talks about the economy or any economic activities (e.g., job losses, economic hardship, economic stalemate)	
11	V1.11	11=TopicPRESENT: Other category	0=No/1=Yes, specify	Yes if the topic is not covered in the categories above,please specify	
12	V1a.1	1=MainTopicONLY: Vaccine	0=No/1=Yes	Yes if the post talks about vaccines, no matter the position taken	
13	V1a.2	2=MainTopicONLY: Masks	0=No/1=Yes	Yes if the post talks about mask wearing, mask safety, or anything about marks, no matter the position taken	
14	V1a.3	3=MainTopicONLY: Lockdown	0=No/1=Yes	Yes if the post talks about lockdown, no matter the position taken	
15	V1a.4	4=MainTopicONLY: Social distancing	0=No/1=Yes	Yes if the post talks about social distancing, no matter the position taken	
16	V1a.5	5=MainTopicONLY: Disease contagion/spread/extent	0=No/1=Yes	Yes if the post talks about how widespread covid is, such as by making reference to pandemic, or how easy/difficult it is to chatch it, no matter the position taken	
17	V1a.6	6=MainTopicONLY: Disease consequences	0=No/1=Yes	Yes if the post talks about disease consequences on self, society, medical system, no matter the position taken	

18	V1a.7	7=MainTopicONLY: Disease cure	0=No/1=Yes	Yes if the post advocates a cure	
19	V1a.8	8=MainTopicONLY: Testing	0=No/1=Yes	Yes if the post is about tests or testing	
20	V1a.9	9=MainTopicONLY: Technology-related	0=No/1=Yes	Yes if the post is about technology (e.g., 5G, chips)	
21	V1a.10	10=MainTopicONLY: Economy-related	0=No/1=Yes	Yes if the posts talks about the economy or any economic activities (e.g., job losses, economic hardship, economic stalemate)	
22	V1a.11	11=MainTopicONLY: Other category	0=No/1=Yes, specify	Yes if the topic is not covered in the categories above,please specify	
23	V1a.12	12=Main topic expressed verbally	0=No/1=Yes	Yes if the main topic is expressed using words	
24	V1a.13	13=Main topic expressed visually	0=No/1=Yes	Yes if the main topic is expressed through visual elements	
25	V2.1	1=ProblemPRESENT: Death	0=No/1=Yes	Yes if there's any mention of death, victims, bodies etc.	
26	V2.2	2=ProblemPRESENT: Ilness	0=No/1=Yes	Yes if there's any mention of illness, disease etc.	
27	V2.3	3=ProblemPRESENT: Dehumanizing	0=No/1=Yes	Yes if there's any mention of changes to the body or mind that are do not occur naturally in humans, making them more like animals or like robots	
28	V2.4a	4a=ProblemPRESENT: Freedom of movement	0=No/1=Yes	Yes if it has to do with people's freedom of doing something/moving	
29	V2.4b	4b=ProblemPRESENT: Freedom of speech/expression	0=No/1=Yes	Yes if it has to do with people's freedom of publicly expressing their opinions, criticism	

30	V2.5	5=ProblemPRESENT: Truth	0=No/1=Yes	Yes if it cleary challenges official accounts of reality (e.g., they say so, but that's not true)	
31	V2.6	6=ProblemPRESENT: Big Brother control	0=No/1=Yes	Yes, if the post complains about state/authorities/elite control over people's decisions, life, movement etc.	
32	V2.6a	6a=ProblemPRESENT: Financial/Economic loss	0=No/1=Yes	Yes if the post talks about personal, collective, local, national or international economic losses, such as economic downtursn, but also job losses	
33	V2.7	7=ProblemPRESENT: Other	0=No/1=Yes, specify	Anything else that doesn't fit in the categories above.	
34	V2a.1	1=PrimaryProblemONLY: Death	0=No/1=Yes	Yes if there's any mention of death, victims, bodies etc.	
35	V2a.2	2=PrimaryProblemONLY: Ilness	0=No/1=Yes	Yes if there's any mention of illness, disease etc.	
36	V2a.3	3=PrimaryProblemONLY: Dehumanizing	0=No/1=Yes	Yes if there's any mention of changes to the body or mind that are do not occur naturally in humans, making them more like animals or like robots	
37	V2a.4a	4a=PrimaryProblemONLY: Freedom of movement	0=No/1=Yes	Yes if it has to do with people's freedom of doing something/moving	
38	V2a.4b	4b=PrimaryProblemONLY: Freedom of speech/expression	0=No/1=Yes	Yes if it has to do with people's freedom of publicly expressing their opinions, criticism	

39	V2a.5	5=PrimaryProblemONLY: Truth	0=No/1=Yes	Yes if it cleary challenges official accounts of reality (e.g., they say so, but that's not true)	
40	V2a.6	6=PrimaryProblemONLY: Big Brother control	0=No/1=Yes	Yes, if the post complains about state/authorities/elite control over people's decisions, life, movement etc.	
41	V2a.6a	6a=PrimaryProblemONLY: Financial/Economic loss	0=No/1=Yes	Yes if the post talks about personal, collective, local, national or international economic losses, such as economic downtursn, but also job losses	
42	V2a.7	7=PrimaryProblemONLY: Other	0=No/1=Yes, specify	Anything else that doesn't fit in the categories above.	
43	V2a.8	8=Primary problem expressed verbally	0=No/1=Yes	Yes if the primary problem is expressed using words	
44	V2a.9	9=Primary problem expressed visually	0=No/1=Yes	Yes if the primary problem is expressed through visual elements	
45	V3.1	1=Blamed: Is someone/something cleary identified as to blame?	0=No/1=Yes	Yes if someone or something is specifically indentified as to blame for the problem	
46	V3.2	2=Blamed: Identified individual	0=No/1=Yes, specify	If yes, give name	
47	V3.3	3=Blamed: Identified group/institution/organization	0=No/1=Yes, specify	If yes, give name	
48	V3.5	5=Blamed: Other category	0=No/1=Yes, specify	Yes if the person/entity/group etc does not fit the categories above. Please specify.	
49	V3.2.a	2.a=Blamed Individual: Politician	0=No/1=Yes	Yes if a politician	

50	V3.2.b	2.b=Blamed Individual: Businessperson	0=No/1=Yes	Yes if the person is connected to business (e.g., Bill Gates)	
51	V3.2.c	2.c=Blamed Individual: Scientist	0=No/1=Yes	Yes if the peson is a scientist	
52	V3.2.d	2.d=Blamed Individual: Other, specify	0=No/1=Yes, specify	Yes if the person is not covered by any of the job categories above.	
53	V3.3.a	3.a=Blamed Group: Government/National authorities	0=No/1=Yes	Yes if the group in question is identified as the government/the authorities	
54	V3.3.b	3.b=Blamed Group: Non-governmental national authority	0=No/1=Yes	Yes if the group is non-governemental but has some advisory/policy authority	
55	V3.3.c1	3.c1=Blamed Group: International organization	0=No/1=Yes	Yes if it's an international group	
56	V3.3.c2	3.c2=Blamed Group: Private company	0=No/1=Yes	YES IF IT'S A COMMERCIAL COMPANY, BUT NOT A TRADITIONAL MEDIA ORGANIZATION, SEE BELOW (e.g. YES FOR Microsoft, Google, Facebook, BUT NO FOR CNN)	
57	V3.3.d	3.d=Blamed Group: Party	0=No/1=Yes	Yes if it's a party	
58	V3.3.d1	3.d=Blamed Group: Media organization	0=No/1=Yes	Yes if a media organization or the media as a group are identified as to blame. CODE YES FOR THE UNSPECIFIED 'MEDIA' OR FOR INDIVIDUAL ORGANIZATIONS, E.G. 'CNN'	
59	V3.3.e	3.e=Blamed Group: Ethnic, sexual, political or religious minority	0=No/1=Yes, specify	Yes if it's a minority of some kind	

60	V3.3.f	3.f=Blamed Group: Scientists as a community	0=No/1=Yes	Yes if it's the scientific community	
61	V3.3.g	3.g=Blamed Group: Other, specify	0=No/1=Yes	Yes, if the group description does not fit any of the categories above. Please specify.	
62	V3.7	7=Blame expressed verbally	0=No/1=Yes	Yes if the blame is expressed using words	
63	V3.8	8=Blame expressed visually	0=No/1=Yes	Yes if the blame is expressed through visual elements	
64	V4.1	1=Cause: Is the cause specified?	0=No/1=Yes	Yes if there's a specific explanation why the problem is occuring	
65	V4.7	7=Cause expressed verbally	0=No/1=Yes	Yes if the cause is expressed using words	
66	V4.8	8=Cause expressed visually	0=No/1=Yes	Yes if the cause is expressed through visual elements	
67	V5.1	1=Solution: Is a solution proposed to deal with the problem?	0=No/1=Yes, specify	Yes, if a solution to a problem is specifically identified. It's not necessary that the problem be also explicitely mentioned, it may be that the problem is simply implied, and the post just goes for the solution.	
68	V5.12	12=Solution expressed verbally	0=No/1=Yes	Yes if the solution is expressed using words	
69	V5.13	13=Solution expressed visually	0=No/1=Yes	Yes if the solution is expressed through visual elements	
70	V6.1	1=VerbalONLY: Human exemplars	0=No/1=Yes	Yes if the post relays ordinary people's experiences, through quotes or just discourse NOTE: IF THEY ARE REPRESENTING A JOB	

				CATEGORY, SUCH AS NURSES OR DOCTORS, THEY DO NOT COUNT AS HUMAN EXEMPLARS	
71	V6.2	2=VerbalONLY: Statistics/Hard data/Numbers	0=No/1=Yes	Yes if the post reports numbers, statistics but not other facts not involving numbers	
72	V6.3	3=VerbalONLY: Quotes	0=No/1=Yes	Yes if the post quotes someone.	
73	V6.4	4=VerbalONLY: Quotes/paraphrases politicians	0=No/1=Yes, specify who	Yes if the post relates an utterance from one/several politicians, whether by quoting them or by 's/he said/claimed that...'	
74	V6.5a	5a=VerbalONLY: Quotes/paraphrases individual experts	0=No/1=Yes, specify who	Yes if the post relates an utterance from one/several officially accepted experts, whether by quoting them or by 's/he said/claimed that...'	
75	V6.5b	5b=VerbalONLY: Quotes/paraphrases individual contested experts	0=No/1=Yes, specify who	Yes if the post relates an utterance from one/several self-styled/contested experts, whether by quoting them or by 's/he said/claimed that...'	
76	V6.6	6=VerbalONLY: Quotes/paraphrases institutions	0=No/1=Yes, specify who	Yes if the post relates an utterance from one/several institutions, whether by quoting them or by 'they said/claimed that...'	
77	V6.7	7=VerbalONLY: Quotes/paraphrases other well-know figure	0=No/1=Yes, specify who	Yes if the post relates an utterance from one/several well-know figures that do not fit the above categories.	

78	V6.8	8=VerbalONLY: Techonology	0=No/1=Yes	Yes if the post make reference to technology, whether in relation to some industry or some product that requires sophisticated equipment to be produced	
79	V6.9	9=VerbalONLY: Pop culture	0=No/1=Yes	Yes if the post make reference to anything cultural - movies, books, music, popular wisdom	
80	V8.1	1=VerbalONLY: Hope	0=No/1=Yes	Yes if there is any adjective or adverb indicating hope or optimism	
81	V8.2	2=VerbalONLY: Relief	0=No/1=Yes	Yes if there is any adjective or adverb indicating relief or happiness	
82	V8.3	3=VerbalONLY: Gratitude	0=No/1=Yes	Yes if there is any adjective or adverb indicating gratitude	
83	V8.4	4=VerbalONLY: Other positive emotion	0=No/1=Yes, specify	Yes if there are any other words indicating positive emotions that are not included in the previous variables. Please specify	
84	V8.5	5=VerbalONLY: Anxiety	0=No/1=Yes	Yes if there is any adjective or adverb indicating anxiety, fear, trepidation, pessimism	
85	V8.6	6=VerbalONLY: Anger	0=No/1=Yes	Yes if there is any adjective or adverb indicating anger, outrage, annoyance	
86	V8.7	7=VerbalONLY: Sadness	0=No/1=Yes	Yes if there is any adjective or adverb indicating sadness, feeling of loss, despair	

87	V8.8	8=VerbalONLY: Other negative emotion	0=No/1=Yes, specify	Yes if there are any other words indicating negative emotions that are not included in the previous variables. Please specify	
88	V8.9	9=VerbalONLY: Profanity	0=No/1=Yes	Yes if therere's any swearing	
89	V10.1	1=VerbalONLY: Need for care and protection	0=No/1=Yes, specify	Yes if the post mentions the importance of caring and helping protect	
90	V10.2	2=VerbalONLY: Need for fairness	0=No/1=Yes, specify	Yes if the post mentions the importance of fairness, treating everybody as one should	
91	V10.3	3=VerbalONLY: Respect of the rules	0=No/1=Yes, specify	Yes if the post mentions the importance of everybody respecting the rules in place (behaving according to the rules)	
92	V10.4	4=VerbalONLY: Loyalty to one's people/country/community	0=No/1=Yes, specify	Yes if the post mentions the importance of solidarity, taking a hit for the greater good of the community	
93	V7.0	1=More than one image in the post?	0=No/1=Yes	If yes, code as many images as needed, delete the variables for the remaining unexisting images.	
94	V7.1	1=FIRST_PIC_VisualONLY: Human exemplars	0=No/1=Yes	Yes if the post shows ordinary people's experiences NOTE: IF THEY ARE REPRESENTING A JOB CATEGORY, SUCH AS NURSES OR DOCTORS, THEY DO NOT COUNT AS HUMAN EXEMPLARS	

95	V7.2	2=FIRST_PIC_VisualONLY: Statistics/Hard data	0=No/1=Yes	Yes if the post shows numbers, statistics, graphs, data	
96	V7.3	3=FIRST_PIC_VisualONLY: Shows politicians	0=No/1=Yes, specify	Yes if the post shows one or several politicians	
97	V7.4a	4a=FIRST_PIC_VisualONLY: Shows individual experts	0=No/1=Yes, specify	Yes, if the post shows one or several officially accepted experts	
98	V7.4b	4b=FIRST_PIC_VisualONLY: Shows contested individual experts	0=No/1=Yes, specify	Yes, if the post shows one or several self-styled/contested experts	
99	V7.5	5=FIRST_PIC_VisualONLY: Shows institution	0=No/1=Yes, specify	Yes if the post shows the symbols associated with institutions (e.g., FLAGS, LOGOS)	
100	V7.6	6=FIRST_PIC_VisualONLY: Shows other well-know figure	0=No/1=Yes, specify	Yes if the post shows some other well-known figure	
101	V7.7a	7a=FIRST_PIC_VisualONLY: Techonology (not medical)	0=No/1=Yes	Yes if the post shows technology, whether in relation to some industry or some product that requires sophisticated equipment to be produced	
102	V7.7b	7b=FIRST_PIC_VisualONLY: Medical equipment	0=No/1=Yes	Yes if the post shows syringes, vials, beds, oxygen etc.	
103	V7.7c	7c=FIRST_PIC_VisualONLY: Medical personnel	0=No/1=Yes	Yes if the post shows nurses or doctors	
104	V7.8	8=FIRST_PIC_VisualONLY: Pop culture	0=No/1=Yes	Yes if the post shows anything cultural - movies, books, music, popular wisdom	
105	V7.9	9=FIRST_PIC_VisualONLY: Shows (symbols of) death	0=No/1=Yes	Yes if the post shows dead, coffins, skeletons…	
106	V7.10	10=FIRST_PIC_VisualONLY: Shows outdoor environment	0=No/1=Yes	Yes if the post shows a picture of the outside (e.g., street, nature etc). IF NOT CLEAR CODE NO	

107	V7.11	11=FIRST_PIC_VisualONLY: Shows indoor environment	0=No/1=Yes	Yes if the post shows the inside of a building (room, hall etc). IF NOT CLEAR CODE NO	
108	V7.12	12=FIRST_PIC_VisualONLY: Other visual element not coded for above	0=No/1=Yes, specify	Yes if there's anything else that is important but not covered by the above	
109	V9.1	1=FIRST_PIC_VisualONLY: Positive emoji	0=No/1=Yes	Yes if there's any clearly positive emoji	
110	V9.2	2=FIRST_PIC_VisualONLY: Negative emoji	0=No/1=Yes	Yes if there's any clearly negative emoji	
111	V9.3	3=FIRST_PIC_VisualONLY: Other emoji	0=No/1=Yes, specify	Yes if there's any other emoji not easy to tell whether it's positive or negative	
112	V9.4	4=FIRST_PIC_VisualONLY: Person visible	0=No/1=Yes	Yes if there's someone shown	
113	V9.5a	5a=FIRST_PIC_VisualONLY: More than one person shown	0=No/1=Yes	Yes if there are several individuals who appear in the post (do not count profile pictures)	
114	V9.5b	5b=FIRST_PIC_VisualONLY: How many people shown?	Count	If more than 15, put 99	
115	V9.6	6=FIRST_PIC_VisualONLY: Face fully visible	0=No/1=Yes	Yes if you can see all their face (no mask on)	
116	V9.7	7=FIRST_PIC_VisualONLY: Face ONLY partially visible	0=No/1=Yes	Yes if you can only see part of their face, including when they are wearing a mask	
117	V9.8	8=FIRST_PIC_VisualONLY: Facial positive expression	0=No/1=Yes	Yes if they display some kind of positive facial expression (smiling, happy, relieved, hopeful etc.)	
118	V9.8a	8a=FIRST_PIC_VisualONLY: Positive expression: happiness	0=No/1=Yes	Yes if they display happiness	
119	V9.9	9=FIRST_PIC_VisualONLY: Facial negative expression	0=No/1=Yes	Yes if they display some kind of negative facial expression (crying, showing ager, fear, disgust etc.)	
120	V9.10	10=FIRST_PIC_VisualONLY: Negative expression: anxiety	0=No/1=Yes	Yes if they show fear or anxiety. If you	

				can't tell, choose No=0	
121	V9.11	11=FIRST_PIC_VisualONLY: Negative expression: anger	0=No/1=Yes	Yes if they show anger or outrage. If you can't tell, choose No=0	
122	V9.12	12=FIRST_PIC_VisualONLY: Negative expression: disgust	0=No/1=Yes	Yes if they show disgust. If you can't tell, choose No=0	
123	V9.13	13=FIRST_PIC_VisualONLY: Negative expression: other, specify	0=No/1=Yes, specify	Yes if they show any other negative emotion, please specify. If you can't tell, choose No=0	
124	V9.14	14=FIRST_PIC_VisualONLY: Eye contact	0=No/1=Yes	Yes if the person shown looks directly in the camera	
125	V9.15a	15a=FIRST_PIC_VisualONLY: Expansive body posture	0=No/1=Yes	Yes if a person takes more space or has limbs extended from the body (e.g., raised arms, hands gesticulating away from the body, or legs spread when seating or standing).	
126	V9.15b	15b=FIRST_PIC_VisualONLY: Contractive body posture	0=No/1=Yes	Yes someone keeps limbs close to the body	
127	V9.16	16=FIRST_PIC_VisualONLY: Profanity	0=No/1=Yes	Yes if there's any rude gesture display.	
128	V11.1	1=FIRST_PIC_VisualONLY: Need for care and protection	0=No/1=Yes, specify	Yes if the post shows someone hurt, in pain, ill, vulnerable or in need of protection	
129	V11.2	2=FIRST_PIC_VisualONLY: Need for fairness	0=No/1=Yes, specify	Yes if the post shows anything indicating fair treatment (through collage? Ovelapping images?)	
130	V11.3	3=FIRST_PIC_VisualONLY: Respect of the rules	0=No/1=Yes, specify	Yes if the post shows mask wearing, vaccine taking, social distancing, any proof of respecting the rules	

131	V11.4	4=FIRST_PIC_VisualONLY: Loyalty to one's people/country/community	0=No/1=Yes, specify	Yes if the post shows solidarity, taking a hit for the greater good of the community, including displaying community signs -- rainbows flags, NHS symbol in the UK	

Note: Variables corresponding to number 94-131 were repeated for every image in the disinformation message.

2. Framing Variables Distributions

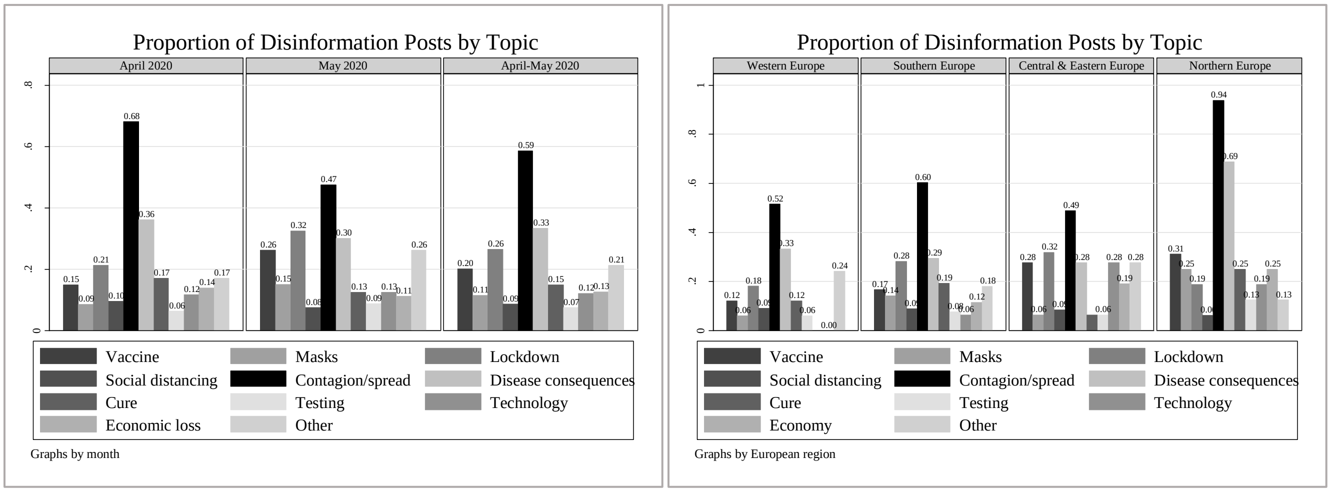

Supplementary Figure 1. Proportion of Disinformation Messages by Topic (Left: by Month, Right: by Region)

Note. Significant differences in the distributions are discussed in the main text.

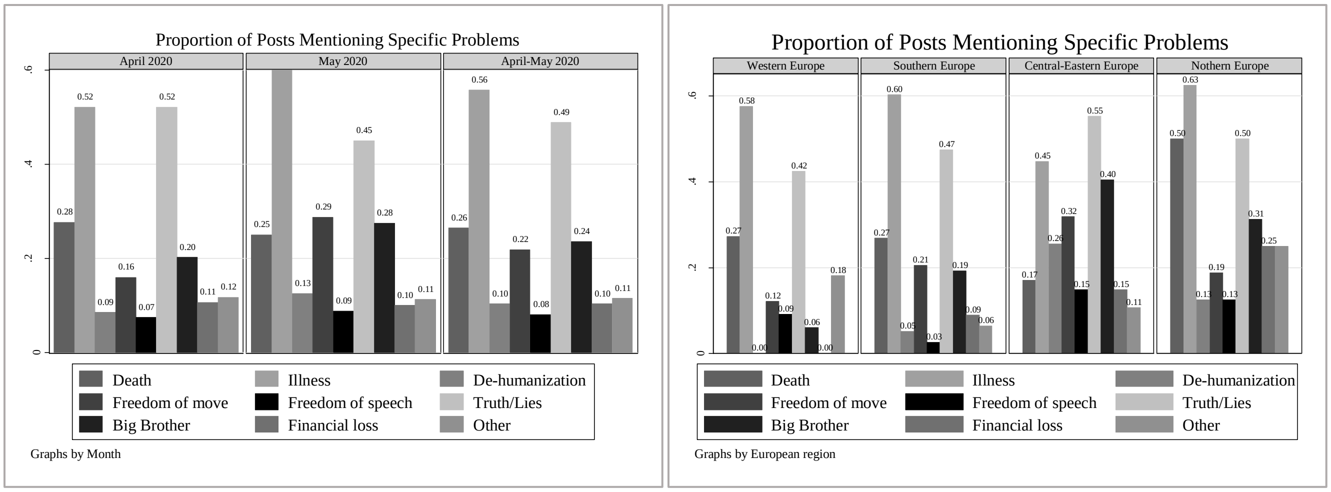

Supplementary Figure 2. Proportion of Disinformation Messages Mentioning Specific Problems (Left: by Month, Right: by Region)

Note. Significant differences in the distributions are discussed in the main text.

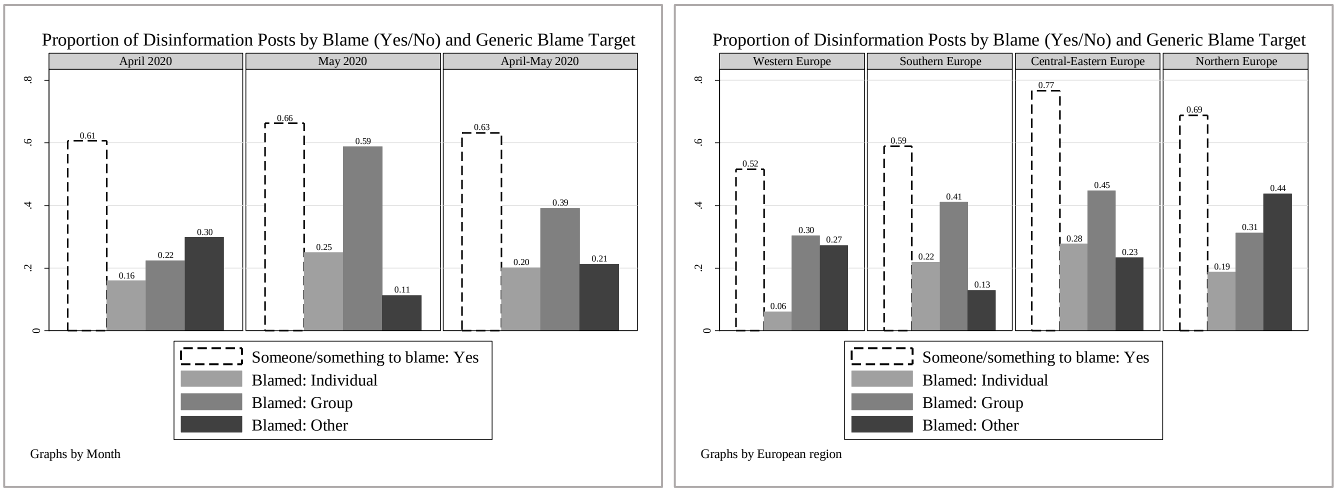

Supplementary Figure 3. Proportion of Disinformation Messages by Blame (Yes/No) and Generic Blame Targets (Left: by Month, Right: by Region)
Note. Significant differences in the distributions are discussed in the main text.

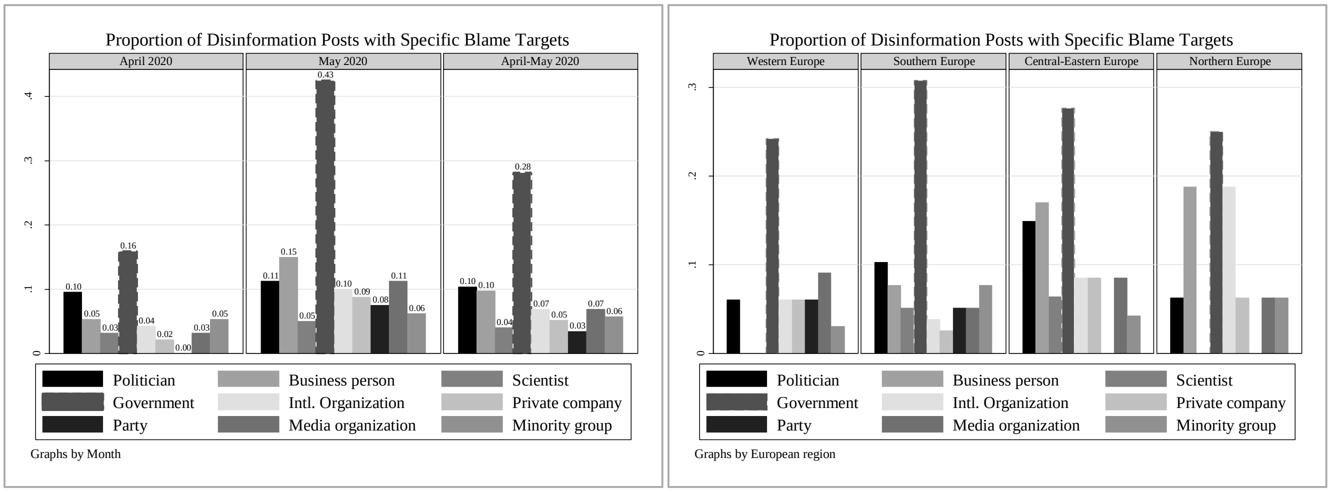

Supplementary Figure 4. Proportion of Disinformation Messages with Specific Blame Targets (Left: by Month, Right: by Region)
Note. Significant differences in the distributions are discussed in the main text.

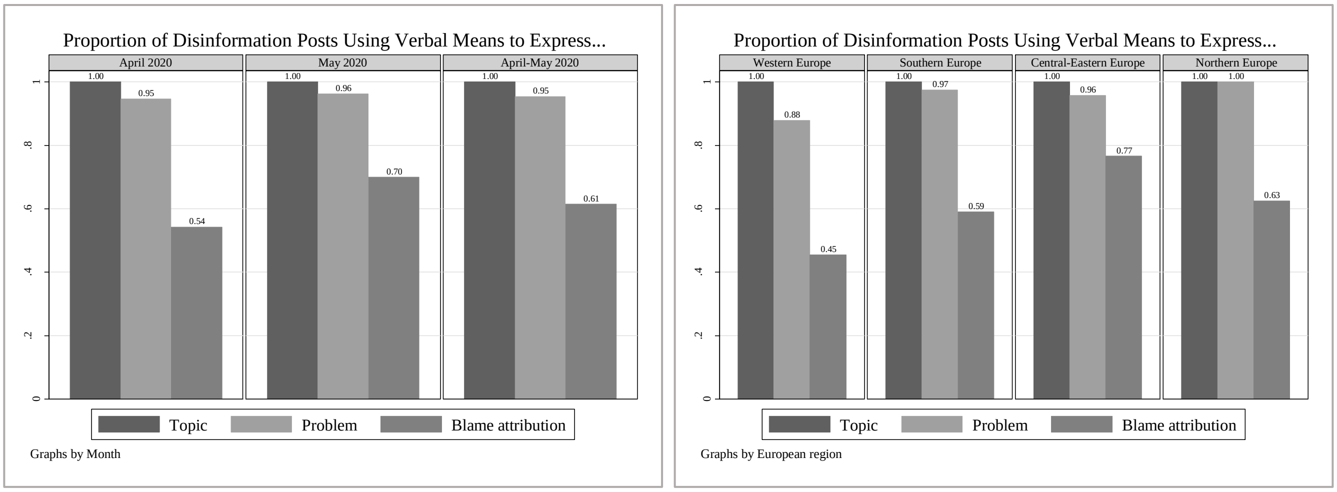

Supplementary Figure 5. Proportion of Disinformation Messages using Verbal Means to Express Different Framing Elements (Left: by Month, Right: by Region)
Note. Significant differences in the distributions are discussed in the main text.

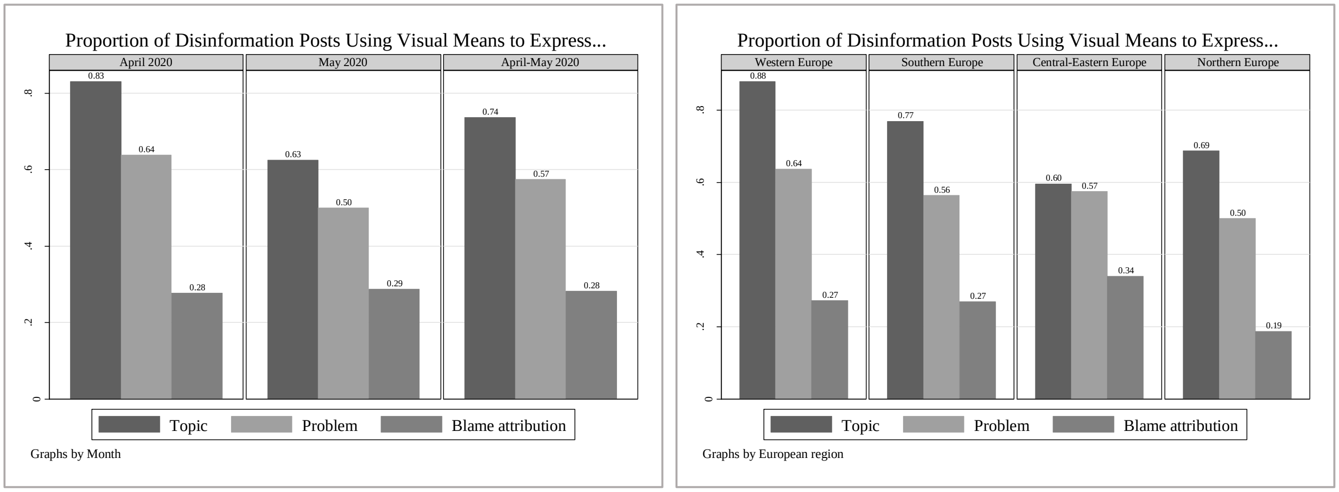

Supplementary Figure 6. Proportion of Disinformation Messages Using Visual Means to Express Different Framing Elements (Left: by Month, Right: by Region)
Note. Significant differences in the distributions are discussed in the main text.

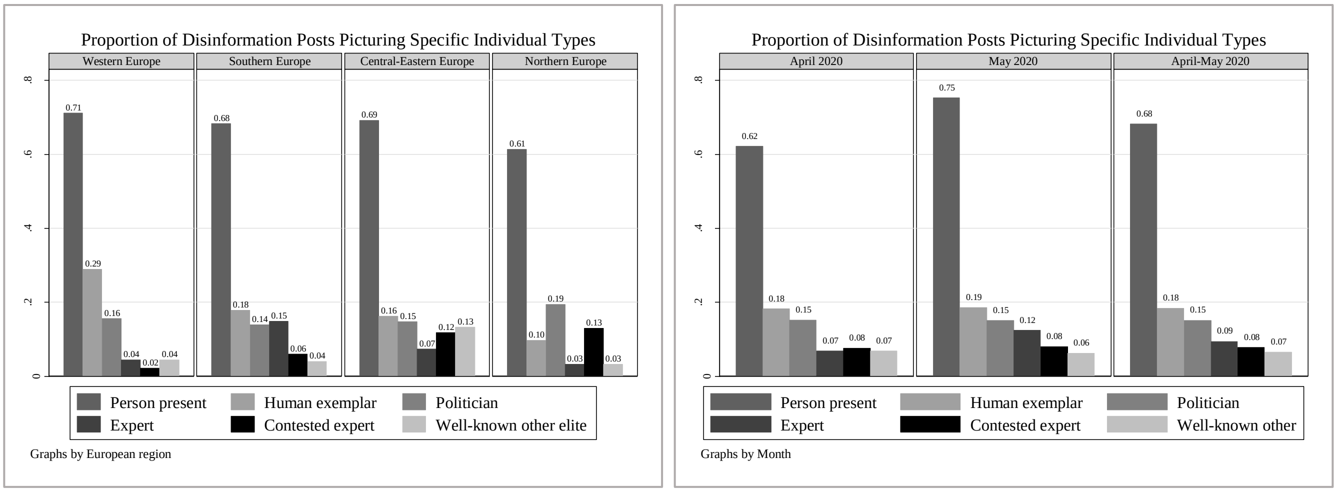

Supplementary Figure 7. Proportion of Disinformation Messages Picturing Specific Individual Types (Left: by Month, Right: by Region)
Note. Significant differences in the distributions are discussed in the main text.

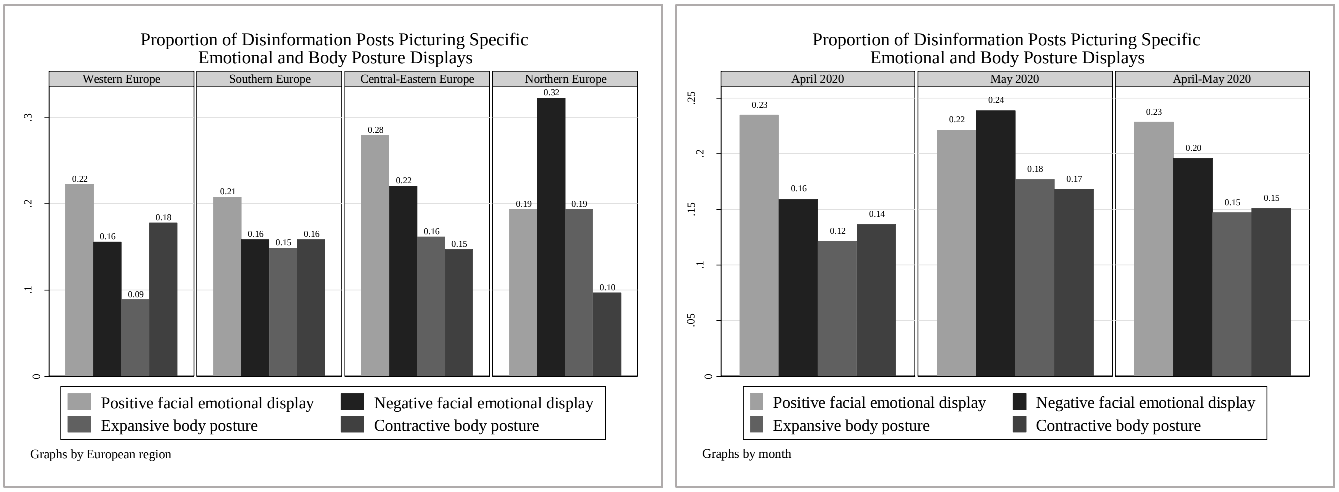

Supplementary Figure 8. Proportion of Disinformation Messages Picturing Specific Emotional and Body Posture Displays (Left: by Month, Right: by Region)
Note. Significant differences in the distributions are discussed in the main text.

3. Additional Tables

Supplementary Table A1. Nonverbal Displays and Generic Blame Targets

	Model A1-1. Dependent Variable: Positive Facial Emotional Display (Yes/No)					Model A1-2. Dependent Variable: Negative Facial Emotional Display (Yes/No)				
	Observed Coef.	Bias	Bootstrap Std. Err.	[95% BCA Conf. Interval]		Observed Coef.	Bias	Bootstrap Std. Err.	[95% BCA Conf. Interval]	
Blamed: Individual	1.094	0.063	0.420	0.258	1.879	0.257	-0.005	0.408	-0.539	1.035
Blamed: Group/ institution/organization	0.781	0.014	0.392	-0.003	1.495	1.095	0.025	0.392	0.276	1.835
Blamed: Other	0.486	0.011	0.457	-0.445	1.351	0.772	0.011	0.489	-0.197	1.792
Constant	-1.976	-0.053	0.342	-2.575	-1.308	-2.168	-0.034	0.348	-2.945	-1.568
N	245					245				
Replications	1500					1500				
Wald chi2(9)	12.77					8.94				
Prob > chi2	0.005					0.030				
Pseudo R2	0.063					0.041				
Log pseudolikelihood	-123.38					-116.231				
Replications based on 174 clusters in id										

	Marginal Effects on P(Positive Facial Emotional Display)					Marginal Effects on P(Negative Facial Emotional Display)				
	Average marginal effect	Bootstrap Std. Err.	P>|z|	[95% Conf. Interval]		Average marginal effect	Bootstrap Std. Err.	P>|z|	[95% Conf. Interval]	
Blamed: Individual	0.210	0.087	0.016	0.039	0.382	0.039	0.062	0.529	-0.082	0.160
Blamed: Group/ institution/organization	0.134	0.069	0.051	-0.001	0.268	0.166	0.056	0.003	0.055	0.276
Blamed: Other	0.084	0.080	0.298	-0.074	0.241	0.117	0.074	0.112	-0.027	0.261

Supplementary Table B1. Nonverbal Displays and Individual Blame Targets

	Model B1-1. Dependent Variable: Negative Facial Emotional Display (Yes/No)					Model B1-2. Dependent Variable: Positive Facial Emotional Display (Yes/No)				
	Observed Coef.	Bias	Bootstrap Std. Err.	[95% BCA Conf. Interval]		Observed Coef.	Bias	Bootstrap Std. Err.	[95% BCA Conf. Interval]	
Blamed Individual: Politician	1.173	0.075	0.477	0.276	2.046	-0.164	-0.022	0.559	-1.210	0.913
Blamed Individual: Businessperson	1.250	0.016	0.419	0.481	2.100	1.114	0.011	0.414	0.297	1.889
Blamed Individual: Scientist	0.853	-0.009	0.792	-1.043	2.276	0.148	0.015	0.727	-1.096	1.815
Constant	-1.622	-0.018	0.205	-2.034	-1.240	-1.552	-0.006	0.190	-1.963	-1.201
N	245					245				
Replications	1,454					1399				
Wald chi2(9)	18.94					7.720				
Prob > chi2	0.0003					0.052				
Pseudo R2	0.0733					0.024				
Log pseudolikelihood	-122.044					-118.29				

Replications based on 174 clusters in id

	Marginal Effects on P(Positive Facial Emotional Display)					Marginal Effects on P(Negative Facial Emotional Display)				
	Average marginal effect	Bootstrap Std. Err.	P>|z|	[95% Conf. Interval]		Average marginal effect	Bootstrap Std. Err.	P>|z|	[95% Conf. Interval]	
Blamed Individual: Politician	0.188	0.074	0.011	0.044	0.333	-0.025	0.085	0.768	-0.193	0.142
Blamed Individual: Businessperson	0.200	0.062	0.001	0.079	0.322	0.171	0.060	0.005	0.052	0.289
Blamed Individual: Scientist	0.137	0.126	0.279	-0.111	0.385	0.023	0.111	0.839	-0.196	0.241

Supplementary Table C1. Nonverbal Displays and Group Blame Targets

	Model C1-1. Dependent Variable: Positive Facial Emotional Display (Yes/No)					Model C1-2 Dependent Variable: Negative Facial Emotional Display (Yes/No)				
	Observed Coef.	Bias	Bootstrap Std. Err.	[95% BCA Conf. Interval]		Observed Coef.	Bias	Bootstrap Std. Err.	[95% BCA Conf. Interval]	
Blamed group: Government	0.819	0.029	0.382	0.041	1.514	0.833	0.054	0.430	-0.177	1.557
Blamed Group: International organization	-0.022	-0.060	0.643	-1.386	1.113	-0.013	0.014	0.760	-1.754	1.235
Blamed Group: Private company	0.643	0.090	0.833	-1.008	2.257	2.099	0.105	0.816	0.174	3.476
Blamed Group: Party	0.705	0.063	0.928	-0.615	2.161	-0.169	0.100	1.145	-1.590	2.276
Blamed Group: Media organization	-0.813	-0.134	0.931	-2.222	0.712	0.004	-0.028	0.975	-1.598	1.186
Blamed Group: Ethnic, sexual, political or religious minority	-0.050	-0.091	0.710	-1.343	1.621	0.295	-0.021	0.652	-0.934	1.762
Constant	-1.485	-0.024	0.215	-1.892	-1.078	-1.843	-0.043	0.240	-2.271	-1.365
N	245					245				
Replications	1,106					1168				
Wald chi2(9)	7.6					11.45				
Prob > chi2	0.267					0.076				
Pseudo R2	0.032					0.060				
Log pseudolikelihood	-127.45					-113.952				

Replications based on 174 clusters in id

	Marginal Effects on P(Positive Facial Emotional Display)					Marginal Effects on P(Negative Facial Emotional Display)				
	Average marginal effect	Bootstrap Std. Err.	P>|z|	[95% Conf. Interval]		Average marginal effect	Bootstrap Std. Err.	P>|z|	[95% Conf. Interval]	
Blamed group: Government	0.152	0.076	0.044	0.004	0.300	0.122	0.061	0.046	0.002	0.242
Blamed Group: International organization	-0.004	0.108	0.973	-0.216	0.208	-0.002	0.111	0.987	-0.220	0.217
Blamed Group: Private company	0.125	0.179	0.486	-0.226	0.475	0.308	0.112	0.006	0.087	0.528
Blamed Group: Party	0.139	0.204	0.494	-0.260	0.539	-0.025	0.168	0.883	-0.354	0.304
Blamed Group: Media organization	-0.115	0.104	0.269	-0.318	0.089	0.001	0.143	0.997	-0.280	0.281
Blamed Group: Ethnic, sexual, political or religious minority	-0.008	0.118	0.944	-0.239	0.223	0.043	0.095	0.650	-0.143	0.230

Supplementary Table A2. Nonverbal Displays and Visual Expression of Framing Components

	Model A2-1. Dependent Variable: Positive Facial Emotional Display (Yes/No)					Model A2-2. Dependent Variable: Negative Facial Emotional Display (Yes/No)				
	Observed Coef.	Bias	Bootstrap Std. Err.	[95% BCA Conf. Interval]		Observed Coef.	Bias	Bootstrap Std. Err.	[95% BCA Conf. Interval]	
Topic expressed visually	-0.515	-0.017	0.390	-1.277	0.270	-1.096	-0.024	0.386	-1.800	-0.263
Problem expressed visually	-0.449	0.028	0.364	-1.230	0.261	-0.361	0.003	0.363	-1.077	0.327
Blame expressed visually	0.856	0.017	0.368	0.090	1.550	0.041	-0.005	0.381	-0.777	0.754
Constant	-0.893	-0.037	0.323	-1.544	-0.295	-0.485	-0.006	0.290	-1.117	0.064
N	245					245				
Replications	1500					1500				
Wald chi2(9)	6.53					12.68				
Prob > chi2	0.088					0.005				
Pseudo R2	0.035					0.053				
Log pseudolikelihood	-127.14					-114.84				
Replications based on 174 clusters in id										

	Marginal Effects on P(Positive Facial Emotional Display)					Marginal Effects on P(Negative Facial Emotional Display)				
	Average marginal effect	Bootstrap Std. Err.	P>|z|	[95% Conf. Interval]		Average marginal effect	Bootstrap Std. Err.	P>|z|	[95% Conf. Interval]	
Topic expressed visually	-0.087	0.065	0.180	-0.215	0.040	-0.163	0.055	0.003	-0.271	-0.055
Problem expressed visually	-0.076	0.061	0.212	-0.196	0.044	-0.054	0.053	0.312	-0.158	0.050
Blame expressed visually	0.145	0.058	0.013	0.031	0.260	0.006	0.057	0.913	-0.105	0.117

Supplementary Table B2. Nonverbal Displays and Visual Expression of Framing Components

	Model B2-1. Dependent Variable: Expansive Body Pose (Yes/No)					Model B2-2. Dependent Variable: Contractive Body Pose (Yes/No)				
	Observed Coef.	Bias	Bootstrap Std. Err.	[95% BCA Conf. Interval]		Observed Coef.	Bias	Bootstrap Std. Err.	[95% BCA Conf. Interval]	
Topic expressed visually	-0.862	0.009	0.417	-1.693	-0.009	-0.919	-0.011	0.450	-1.801	-0.006
Problem expressed visually	-0.629	-0.035	0.412	-1.396	0.221	0.513	0.059	0.477	-0.481	1.408
Blame expressed visually	0.567	0.012	0.415	-0.231	1.484	-0.147	-0.026	0.497	-1.175	0.778
Constant	-1.027	-0.041	0.325	-1.635	-0.362	-1.369	-0.084	0.395	-2.103	-0.668
N	245					245				
Replications	1500					1500				
Wald chi2(9)	8.16					4.60				
Prob > chi2	0.043					0.204				
Pseudo R2	0.045					0.031				
Log pseudolikelihood	-97.618					-100.775				

Replications based on 174 clusters in id

	Marginal Effects on P(Expansive Body Pose)					Marginal Effects on P(Contractive Body Pose)				
	Average marginal effect	Bootstrap Std. Err.	P>|z|	[95% Conf. Interval]		Average marginal effect	Bootstrap Std. Err.	P>|z|	[95% Conf. Interval]	
Topic expressed visually	-0.104	0.051	0.041	-0.203	-0.004	-0.115	0.057	0.043	-0.225	-0.004
Problem expressed visually	-0.076	0.048	0.118	-0.171	0.019	0.064	0.059	0.282	-0.053	0.181
Blame expressed visually	0.068	0.048	0.159	-0.027	0.163	-0.018	0.062	0.767	-0.139	0.103

Supplementary Table A3. Nonverbal Displays and Type of Individuals Depicted

	Model A3-1. Dependent Variable: Positive Facial Emotional Display (Yes/No)					Model A3-2 Dependent Variable: Negative Facial Emotional Display (Yes/No)				
	Observed Coef.	Bias	Bootstrap Std. Err.	[95% BCA Conf. Interval]		Observed Coef.	Bias	Bootstrap Std. Err.	[95% BCA Conf. Interval]	
Human exemplar	0.520	0.042	0.574	-0.645	1.677	1.100	0.026	0.488	0.083	2.024
Politician	1.760	0.096	0.541	0.612	2.699	1.918	0.063	0.520	0.839	2.851
Expert	1.908	0.054	0.642	0.646	3.144	1.282	0.059	0.591	0.054	2.361
Contested individual expert	1.804	0.055	0.671	0.446	3.062	1.988	0.058	0.614	0.786	3.173
Other well-known elite	2.439	0.102	0.725	0.929	3.760	1.075	0.023	0.779	-0.581	2.590
Constant	-2.281	-0.091	0.369	-2.930	-1.619	-2.472	-0.067	0.338	-3.146	-1.869
N	245					245				
Replications	1493					1491				
Wald chi2(9)	21.66					19.79				
Prob > chi2	0.001					0.001				
Pseudo R2	0.167					0.124				
Log pseudolikelihood	-109.67					-106.170				

Replications based on 174 clusters in id

	Marginal Effects on P(Positive Facial Emotional Display)					Marginal Effects on P(Negative Facial Emotional Display)				
	Average marginal effect	Bootstrap Std. Err.	P>|z|	[95% Conf. Interval]		Average marginal effect	Bootstrap Std. Err.	P>|z|	[95% Conf. Interval]	
Human exemplar	0.074	0.081	0.359	-0.084	0.233	0.150	0.067	0.024	0.020	0.281
Politician	0.251	0.067	0.000	0.119	0.383	0.262	0.064	0.000	0.138	0.387
Expert	0.272	0.083	0.001	0.109	0.435	0.175	0.078	0.025	0.022	0.329
Contested individual expert	0.257	0.089	0.004	0.082	0.432	0.272	0.080	0.001	0.116	0.428
Other well-known elite	0.348	0.089	0.000	0.174	0.521	0.147	0.104	0.158	-0.057	0.351

Supplementary Table B3. Nonverbal Displays and Type of Individuals Depicted

	Model B3-1. Dependent Variable: Expansive Body Pose (Yes/No)					Model B3-2 Dependent Variable: Contractive Body Pose (Yes/No)				
	Observed Coef.	Bias	Bootstrap Std. Err.	[95% BCA Conf. Interval]		Observed Coef.	Bias	Bootstrap Std. Err.	[95% BCA Conf. Interval]	
Human exemplar	0.660	-0.003	0.584	-0.519	1.701	245	245	245	245	245
Politician	1.753	0.052	0.520	0.742	2.788	1500	1500	1500	1500	1500
Expert	1.267	0.060	0.656	-0.374	2.415	8.16	8.16	8.16	8.16	8.16
Contested individual expert	1.115	-0.024	0.866	-0.882	2.663	0.043	0.043	0.043	0.043	0.043
Other well-known elite	1.744	0.082	0.790	-0.261	3.017	0.045	0.045	0.045	0.045	0.045
Constant	-2.694	-0.098	0.368	-3.376	-2.035	-97.618	-97.618	-97.618	-97.618	-97.618
N	245					245				
Replications	1470					1333				
Wald chi2(9)	15.85					10.22				
Prob > chi2	0.007					0.069				
Pseudo R2	0.115					0.061				
Log pseudolikelihood	-90.545					-97.667				

Replications based on 174 clusters in id

	Marginal Effects on P(Expansive Body Pose)					Marginal Effects on P(Contractive Body Pose)				
	Average marginal effect	Bootstrap Std. Err.	P>|z|	[95% Conf. Interval]		Average marginal effect	Bootstrap Std. Err.	P>|z|	[95% Conf. Interval]	
Human exemplar	0.073	0.066	0.264	-0.055	0.202	0.133	0.058	0.021	0.020	0.246
Politician	0.195	0.055	0.000	0.087	0.302	-0.056	0.091	0.539	-0.234	0.122
Expert	0.141	0.072	0.052	-0.001	0.282	0.131	0.073	0.072	-0.012	0.273
Contested individual expert	0.124	0.097	0.202	-0.067	0.314	0.029	0.080	0.715	-0.127	0.185
Other well-known elite	0.194	0.087	0.026	0.024	0.363	0.131	0.092	0.152	-0.048	0.311
